# Evidence on the impact of Baltic Sea ecosystems on human health and well-being: a systematic map

**DOI:** 10.1186/s13750-021-00244-w

**Published:** 2021-11-06

**Authors:** Joanna Storie, Monika Suškevičs, Fiona Nevzati, Mart Külvik, Tinka Kuhn, Benjamin Burkhard, Suvi Vikström, Virpi Lehtoranta, Simo Riikonen, Soile Oinonen

**Affiliations:** 1 Institute of Agricultural and Environmental Sciences, Estonian University of Life Sciences, Kreutzwaldi 1, 51006 Tartu, Estonia; 2 Institute of Physical Geography and Landscape Ecology, Gottfried Wilhelm Leibniz University Hannover, Schneiderberg 50, 30167 Hannover, Germany; 3 Finnish Environment Institute, Latokartanonkaari 11, 00790 Helsinki, Finland

**Keywords:** Evidence synthesis, Participatory approach, Policy relevance, HELCOM region, Ecosystem services, Marine and coastal environment

## Abstract

**Background:**

While the unique marine and coastal environment of the Baltic Sea provides numerous ecosystem services, its ecosystems are under pressure due to the intensification and diversification of anthropogenic uses. This present work constitutes a systematic map of the evidence of the impacts of ecosystem services and disservices on human health and well-being. The aim is to create a better understanding of the threats of unsustainable management or the benefits of sustainable management of the Baltic Sea and the impacts these may have on the health and well-being of human populations and present these findings to policy advisors. The mapping process is described, and the characteristics of the evidence base are presented.

**Methods:**

The applied method has been previously published in a systematic map protocol. Literature searches were carried out in English considering published peer-reviewed literature from traditional scientific journals and scientific reports from the grey literature, using synthesis software. A total of 17 databases were searched. Articles were screened in stages at title and abstract stage, then full-text stage. Geographic limitations were placed on the searches in accordance with research funders call, however, watersheds that had an impact on the Baltic Sea marine and coastal regions were considered. We used the more open PEO format, where population (P) included the human populations within the marine and coastal environment of the Baltic Sea region, exposure (E) related to the Baltic Sea ecosystems services and disservices, and the outcome (O) included all aspects of human health and well-being. After full-text screening articles selected for inclusion were searched for metadata connected to bibliographic information, ecosystem services, health and well-being outcomes and policy relevance.

**Review findings:**

Out of 6456 hits only 460 studies discussed either health or well-being indicators to some extent. Of these, only 67 explicitly mentioned ecosystem services and health and well-being indicators. However, few in this subset engaged with the topic of ecosystem services or disservices and health and well-being in depth. Studies are increasingly relating the two concepts but currently it is mainly studies focussed on cultural ecosystem services that deal with the concept of health and well-being to a greater degree. Studies in the medical literature relating to impacts on health from exposure to the Baltic Sea did not relate their findings to ecosystem services. The database of 67 studies is attached as Additional file 5.

**Conclusions:**

Ecosystem services play an important role in human health and well-being; however, we found few studies that explicitly examine these impacts in detail. Further research is needed to link the health and well-being outcomes from the Baltic Sea to the ecosystem services supplied and therefore to demonstrate the benefits and disservices provided by the Baltic Sea ecosystems to human populations.

**Supplementary Information:**

The online version contains supplementary material available at 10.1186/s13750-021-00244-w.

## Background

The brackish water condition and the enclosed location of the Baltic Sea creates unique ecosystem conditions that make it especially sensitive to the intensification of human uses [1]. This fragile environment supplies a variety of products and recreational services that contribute to the health and well-being of nearly 149 million people that make up the European Union Baltic Sea region countries, as well as the population of the Russian Federation’s Baltic Sea coast [1]. However, these benefits are often inadequately represented as the benefits are difficult to value and intangible [2]. The Baltic Sea has suffered from many well-documented anthropogenic pressures [1] and HELCOM (Baltic Marine Environment Protection Commission), the governing body for the Baltic Sea, has been working to reverse the impacts. The aim was to reach a Good Environmental Status (GES) as defined by the Marine Strategy Framework Directive (MSFD) [3] by 2021 through the development of the Baltic Sea Action Plan (BSAP), adopted in 2007 [4]. The four goals of the BSAP programme are: (1) to reduce eutrophication; (2) reduce the impact of hazardous substances on marine life; (3) ensure environmentally friendly maritime activities and (4) achieve favourable conservation status of Baltic Sea biodiversity. This and other policies with an impact on the Baltic Sea, e.g. the Marine Strategy Framework Directive (2008/56/EC) [3] are listed in Additional file 2.

The goals of the BSAP, however, have not been reached despite leading to encouraging progress in the environmental status of the Baltic Sea. The aim, therefore, was to update the plan in 2021 and to take into consideration new evidence, which is especially pertinent as we head into the decade of the Ocean for Sustainable development [5]. In 2017, the BONUS Secretariat (the legal management organisation of BONUS—joint Baltic Sea research and development programme) called for action to meet these challenges by synthesising the scientific evidence on sustainable uses of the Baltic Sea resources and establishing what gaps in research still need to be addressed [6]. The BONUS ROSEMARIE project: “Blue health and wealth from the Baltic Sea—a participatory systematic review for smart decisions” was one of the projects that arose from that call. The aim of this project was to work in conjunction with experts in marine science and policy advisors to provide the policymakers with the evidence they required through a systematic search of the literature that covered a range of disciplines and feed it back to them. The aim of working with the experts and policy advisors was to ensure the relevance of the project for policy support and improve the credibility and legitimacy of research undertaken in marine science [7].

HELCOM’s work on the environmental status of the Baltic Sea ecosystem embraces a variety of sectors, for example fishing—both commercial and recreational, shipping, tourism, conservation activities, agricultural and sewage and so on that relates to impacts on the Baltic Sea. All these aspects have the potential to impact the health and well-being of people who live, work or visit the Baltic Sea region [8]. Health and well-being is a broad concept that moves beyond an absence of ill-health and encompasses the facets of life that ensure that humans flourish [8]. The natural environment, including the oceans and seas, are essential to human health and well-being by providing the elements that sustain life, from adequate nourishment to the provision of many of the raw materials that provides people with an adequate livelihood free from deprivation. The oceans and seas therefore have an important role to play in contributing to a satisfactory quality of life [5, 9]. The natural environment also provides cultural ecosystem services that plays a role in subjective well-being for example through the aesthetic appreciation of the natural landscape and space for recreational activities in a pleasant environment [2, 10]. Increasing recreational activities also leads to an improvement in physical health as well as mental well-being of individuals [5]. Ignoring these social dynamics, dependent upon ecosystems, risks a failure of the management of the ecosystem service provisions [9]. Health and well-being has multiple dimensions and in this paper we use the domains outlined by McKinnon et al. [11] (see Table 1).

**Table 1:** Domains and definitions of human well-being outcomes [11] (taken from previously published protocol [8])

Domain	Code definition
Economic living standards	Income, employment, employment opportunities, wealth, poverty, savings, payments, loans
Material living standards	Assets owned, access and availability of food, fibre and fuel, basic infrastructure (electricity, water, telecommunications and transportation), shelter
Health	Physical health, nutrition, longevity/life expectancy, maternal health, child health, access to health care, occurrence of diseases, mental health
Education	Education infrastructure (access to school, access to training, quality of education); informal education (transfer of knowledge and skills includes livelihood skills, traditional knowledge and skills); formal education (degrees awarded, students enrolled)
Social relations	Interactions between individuals, within and/or between groups (communities, stakeholders, ethnic groups, gender); conflict, relationships, connectedness, ability to work together, ability to help others, and trust
Security and safety	Physical security (personal safety and security), resource security; tenure security; human rights; vulnerability, resilience and adaptive capacity
Governance (and empowerment)	Structures and processes for decision making, including both formal and informal rules; includes participation and control in decision making, accountability, justice, transparency and governance skills
Subjective well-being	Measures of happiness, quality of life, satisfactions supported by some value of ecosystem(s) and/or resources
Culture and spirituality	Cultural, societal and traditional values of natural resources and nature to the community; sense of home; cultural identity and heritage; spiritual or religious beliefs and/or values
Freedom of choice and action	Ability to pursue what you value doing and being

It has been argued that policies designed to improve the environmental status are also good for the health and well-being of those exposed to the marine environment [5, 12, 13]. Furthermore, Rivero and Villasante [13] argue that research in marine ecosystem services is needed at multiple levels: international, national and local, to examine the specificities of the area and how they are related to the wider ecosystem service provision of marine environments. For example, regulations to reduce coastal disturbances and harmful activities in the sea area are a prerequisite for the sustainable management of fish stocks [5, 14]. In addition, efforts to reduce long-term pollutants have repercussions in reducing potential carcinogens in seafood (for example [5, 15, 16]). These policies, however, need to be based on sound scientific evidence. They also need to consider the various impacts that ecosystems have on people. McKinnon et al.’s domains aims to provide a robust framework to elaborate the linkages between humans and nature. This map therefore aims to use these domains to provide policy advisors with the evidence regarding health and well-being linked to the ecosystem services the Baltic Sea provides and where the gaps are in the research. The evidence for this is distributed amongst a range of sources, much of which is inaccessible to and poorly understood by policy advisors due to its complex nature and hidden behind paywalls [2, 10, 15]. As Stiglitx et al. [17] state, “What we measure affects what we do, and if our measurements are flawed, our decisions may be distorted.” Alongside this, there have been increasing calls for transparent, effective, co-designed research, developed with inputs from practitioners and policy advisors, which measure aspects that matter to people [13, 18, 19]. The advantages of co-designed research are that it brings knowledge from a diverse range of experience and perceptions and improves legitimacy [18, 20, 21]. Policy advisors were approached at the outset of the mapping process to ensure the questions developed were relevant, to understand the level of understanding of the topic and the needs of the policy advisors. Preliminary results were presented to the policy advisors and experts in the field to also ensure the research was presented in a way that was relevant to their needs.

A concept that facilitates better understanding of human-environmental systems is the ecosystem services framework [13]. Ecosystem services are defined as benefits people obtain from nature [22]. The ecosystem service framework links human action, pressures as well as environmental management, and their impact on ecosystem structures and functions. The state of biophysical structures and processes and the resulting ecosystem functions determine the capacities of ecosystems to supply ecosystem services. This ecosystem service ‘cascade’ highlights the interrelations of human activities and anthropogenic pressures, the environmental status and the consequent reciprocal effects on society [13, 23]. Ecosystem services are commonly categorised into provisioning, regulating and cultural ecosystem services. Several classifications (e.g. Millennium Ecosystem Assessment (MEA) [22], Common International Classification of Ecosystem Services (CICES) [24], The Economics of Ecosystems and Biodiversity’ (TEEB) [25]) exist to build common ground to analyse the synergies and trade-offs between different ecosystem services. In addition, the integration of ecosystem services with human well-being concepts has to consider the possible trade-offs between the need to protect ecosystems and the sustainable use of natural resources, thus potentially solving the conflicts between different marine users [12, 26].

However, when discussing the services that ecosystems provide that benefits human health and well-being, it also has to be mentioned that ecosystems can lead to negative impacts on health and well-being [27–29]. These ecosystem disservices are defined by Shackleton et al. as “the ecosystem generated functions, processes and attributes that result in perceived or actual negative impacts on human wellbeing” [30]. Disservices include diseases caused by pathogens and their vectors [29], a recent example being Covid19, and other viruses, bacteria and fungi that cause ill-health and the animals and birds that can act as intermediaries in transmitting them or invasive alien species that threaten commercially exploited populations [28]. These disservices are not restricted to particular places and can occur anywhere in the world where humans and the natural world interact closely [28]. Ecosystem disservices, the negative effects of nature on human well-being, are not limited to pathogens, they also include crop pests, which impact food provision; dangerous animals that can cause injury or death to humans; invasive alien species from plants that change the landscape, to species being a source of new pathogens; allergens and poisonous substances; and dangers from storms, floods and weather events [5, 29, 31, 32]. Dunn [29] and Blanco [27] argue that managing to prevent ecosystem disservices may be more important than managing for ecosystem services by promoting behaviour that reduces harm from ecosystem impacts.

Although the concept of ecosystem services is a fast-growing field in the scientific community, there has been limited incorporation into practise in marine regulations and policies [13]. Armoškaite et al. [26] suggest that the concept has not yet moved on from the theoretical conceptualisation of the supply of ecosystem services. In addition, the understanding of the connections between ecosystem properties and functions and the interrelated supply of ecosystem services are still inadequate, especially in the marine environment [13].

Rivero and Villasante [13] and Fleming et al. [5] suggested that research into marine ecosystem services linkages with health and well-being is particularly needed and understanding of the interplay between the two requires urgent action. As Fleming et al. [5] state the linkages between ecosystem services and human health and well-being need to be made more explicit and one approach is to use ecosystem service assessments that link bio-physical indicators with the ecosystem service provided to humans. These linkages can also inform decision-making by outlining the diverse ways that coastal systems meet the needs of people and the multiple values that people hold of coastal ecosystems [5, 12]. Integrated management approaches are needed to address the challenges faced by those who rely on the ecosystem services provided by the Baltic Sea, for example, through their work or proximity to the sea environment [14, 16]. However, marine policies primarily address the impact of human activities on the environment and not the impact of the ecosystem services on humans [5]. Thus, it is essential that sound scientific evidence is made available to policymakers to formulate policies that consider the important role of the Baltic Sea ecosystem on people [5, 33]. Strengthening the link between ecosystem services and health and well-being management of the Baltic Sea requires cooperation across sectors and evidence from multiple disciplines [24, 25].

## Stakeholder involvement

Various stakeholders have been included at different stages in this mapping process to help generate the questions and test the policy relevance of the results. We mainly targeted stakeholders from the HELCOM GEAR group (Group for the Implementation of the Ecosystem Approach). HELCOM is one of the most important institutions building a science-policy bridge in Baltic Sea governance. Policy advisors have limited time resources to provide continuous input into such searches and therefore linking workshops to conferences and meetings they were involved in, provided a convenient way of gathering their input. Their inputs helped to ensure the policy relevance of the question and how the information can be made accessible for decision making. An initial scoping phase was carried out through three interviews with stakeholders from the HELCOM GEAR group in Estonia, Finland and Sweden and one interview conducted with a stakeholder from the HELCOM GEAR group and an expert for marine environmental economics in Germany. The interviews highlighted that ecosystem services in general and their connection to human health and well-being were not well understood by the policy advisors, despite attempts to integrate these into marine policies in the Baltic Sea area. A following workshop involving 17 participants and an interactive seminar involving 69 participants were used as platforms to elaborate the knowledge gaps and for outlining the preliminary results. In these workshops and seminars, the preliminary results were discussed and helpful feedback was elicited for the presentation of the final results in ways that would be helpful to the policy advisors.

## Objective of the systematic map

The aim of this systematic map is to gather evidence of the impacts on different domains of health and well-being of human populations who are exposed to Baltic Sea ecosystem services. The primary question was, “What linkages have been researched between Baltic Sea ecosystem services and the positive and negative impacts to human health and well-being?” In addition, in accordance with the protocol [8], this question was divided into the following study components using the more open PEO format, where population (P) refers to the human populations within the marine and coastal environment of the Baltic Sea region, exposure (E) to the Baltic Sea ecosystems services and disservices, and outcome (O) refers to aspects of human health and well-being. The open PEO question format is suitable for systematic maps, as it allows the nature and quantity of evidence to be described, rather than answering specifically targeted closed-framed effectiveness-type of questions as systematic reviews do [34–36].

## Methods

This systematic map is based on the methods published in an earlier protocol [8]. The method follows the Collaboration for Environmental Evidence (CEE) Guidelines and Standards for Evidence Synthesis in Environmental Management and this paper follows the ROSES reporting standards [37] (Additional file 1). Below we summarise the main steps taken and describe the changes made compared to the protocol [8] when conducting this systematic map.

### Deviations from the protocol

We deviated from the protocol with respect to the number of languages searched due to time constraints. Searches, therefore, were only conducted in English, which also had certain effect on our pool of articles derived from the databases and search engines. In addition, we did not use the Google search engine—partially due to time constraints set by the duration of the project, but also due the assumption that, according to existing guidelines [38] Google Search is not expected to have a considerable effect on the results. Additionally, we did not search EMBASE as we did not have access to this database via Finnish Environment Institute (SYKE) or Helsinki Library where the searches were carried out. We acknowledge that these deviations imply certain limitations to our results, which we discuss below.

### Search strategy

Searches were conducted between 29.01.2020–06.04.2020. For the searches of subscription-only academic databases, Web of Science (Web of Science Core Collection, Timespan = All years (1975–2020)/LANGUAGE: English), MEDLINE, PUBMED, SCOPUS and CAB Abstracts, we used the subscriptions of SYKE (Finnish Environment Institute) and the Helsinki University Library. The national databases of the project team, Finland, Estonia, Sweden and Germany, were also searched. These databases were considered important by project team members in the respective countries when searching for information on ecosystem services. We adapted and simplified the string for these further respective databases/search platforms or engines (see Additional file 3 for the list).

### Estimating the comprehensiveness of the search

Seven articles of known relevance (benchmark list) were used during the scoping phase to test the search string. The search string was adapted according to test searches if the benchmark papers were not found. However, our final search string did not find two benchmark papers. One benchmark paper was not found due to issues encountered with the ‘KeyWords Plus’ feature in Web of Science. Inclusion of this in the search initially led to a large number of irrelevant studies. Thus, a more restricted search of title and abstract was undertaken to take into account the resources and timing of the project, as detailed in the protocol. This could have led to some bias in the outcome and relevant studies missed, as in the case of the benchmark article, by Ström et al. [39], which lacked geographical information in the title and abstract. The second benchmark article that was not retrieved with the search string, by Piwowarczyk et al. [40]. This needed additional keywords in the search string such as municipalit*, town*, cit*, but when these words were included in the search string too many articles relating to public health in the Baltic countries were returned. These articles, though, did not necessarily relate to issues directly resulting from exposure to the Baltic Sea ecosystems. Similar articles in related studies were found by these authors though. The sample size from the final search string, however, gives important indications as to the focus of research regarding the impact of the Baltic Sea ecosystems on health and well-being.

### Study eligibility criteria

The overall eligibility decision included a stepwise approach with their respective categories listed in Table 2.

**Table 2:** Eligibility criteria and examples of studies excluded

Criteria	Included	Excluded
Geographical coverage	Studies explicitly reporting health and well-being evidence regarding the Baltic Sea basins as defined in the Helsinki Convention [41]	All studies from Germany, Denmark and Sweden West coast that did not specifically mention the Baltic Sea/Kattegat or show on a map a sampling point etc. for a Baltic Sea area Studies related to ticks which did not indicate that ticks were collected from a coastal site Studies with different locations mentioned with minor reference to the Baltic Sea
Population	Human populations exposed to the Baltic Sea ecosystem	Studies which focussed only on populations prior to the 1900s
Exposure	Exposure to the Baltic Sea ecosystems	Studies onboard ships, no interaction with the sea—e.g. nutrition aboard a ship but not those risks/injuries due to exposure to the weather Studies on bycatch and discards, unless specific mention was made of the animals/birds as competitors or in conflict with the fishing industry Studies on marine n-3-fatty acids, chemicals and hazardous substances and associated terms, unless there was specific mention of being derived from fish from the Baltic Sea (or Kattegat) or an activity based in the Baltic Sea
Outcomes	Studies which explicitly assessed impacts on health and well-being	Studies assessed as having Level 1 or 2 with respects to the Level of engagement with health or well-being topics (see below)

**Fig. 1:**
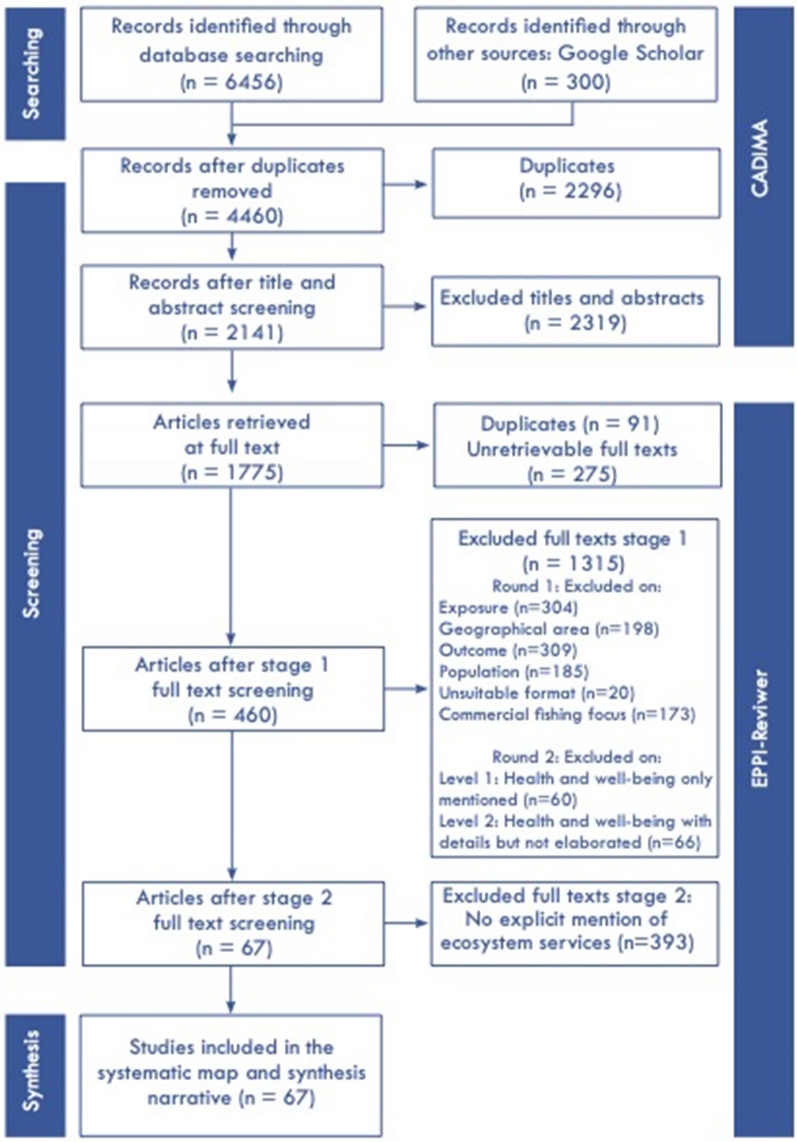
ROSES flowchart of the screening process. The diagram also depicts at what stage the online tools CADIMA and EPPI-reviewer were used. The final studies (n = 67) were selected based on studies that explicitly mentioned ecosystem services and elaborated on health and well-being to some extent. All excluded articles are included in Additional file 4

At the screen on full-text stage it was found that studies varied in the way that the topic of health and well-being was discussed in the text. Some studies mentioned the words “health” and “well-being” but did not further elaborate on this topic, it was therefore necessary to assess their level of engagement with the topic in a further round (see Fig. 1). In Round 2, three levels were used where Level 1 merely mentioned health and/or well-being, for example an article may mention that contamination may affect human health, but there is no further mention of the topic and the focus is on the aquatic flora and fauna. At Level 2, health and well-being details were added to these related to the topic of the study, however, it was only mentioned in the introduction or the discussion or conclusion, for example contamination of fish for human consumption but not related to any specific health effects. At Level 3, mention of health and well-being occurred at least in the introduction and discussion or conclusion and included additional details, such as the specific benefits listed or impacts provided by ecosystem services to health and well-being of the human population. For the purposes of this systematic map, only studies at Level 3 were included in the systematic map. Studies at Levels 1 and 2 were considered insufficient in engaging with the health and well-being aspect (see (Additional file 4: Sheet: Exclusion criteria Round 2). This reduced the number of articles for inclusion from 753 to 460. Only articles explicitly mentioning ecosystem services were then selected from the 460 articles, n = 67. Assigning the remaining 383 articles to the relevant ecosystem services was beyond the scope of this mapping exercise as it requires a degree of interpretation and further resources.

Additionally, studies were excluded for the following pragmatic reasons:

- Although we conducted searches in English, some non-English language results occurred, which we excluded in the screening phase.
- PDF files were not available or inaccessible. It should be noted that at this stage that over 70 PDF files were accessed via Gottfried Wilhelm Leibniz University Hannover as they were not available to SYKE (Finnish Environment Institute) nor the Helsinki University Library. Others were obtained via requests sent through ResearchGate.
- Unsuitable formats for line-by-line coding (such as scanned pictures).
- Presentation slides for conferences and meetings.
- Duplicate studies, for example a case study that was later included in a broader range of case studies, or PhD dissertations based on published articles and vice versa.

A full list of articles and the reason for their exclusion at full-text stage is included in Additional file 4.

### Article screening strategy

Synthesis software was used to screen the articles. Synthesis software supports the gathering, selecting and analysing of literature for reviews or mapping of evidence [42]. Initially, CADIMA a free web-based tool [43] was used, followed by a subscription only software, EPPI-Reviewer [44], as the latter has more advanced functionalities for data coding. Studies were uploaded to CADIMA to be screened at title and abstract level. At least two screeners independently assessed a subset of 5% of all search results (n = 323, Fig. 1). The results from the screening of the 5% subset were compared and disagreements were resolved by discussions among the screeners and the rest of the team where necessary. During this process, we aimed for a Kappa value minimum of 0.7 [8] and in the final outcome we reached 0.74, suggesting our agreement was more than adequate for the review process. It was found, however, that limitations within the CADIMA software to carry out full text screening and data coding, as well as the volume of searches returned, necessitated a move to EPPI-Reviewer (see Fig. 1). Any papers authored by one of the reviewers was screened and coded by one of the other reviewers.

As Kohl et al. explain CADIMA provides a simple clear format for screening of titles and abstracts [42], however the team working on this analysis felt that EPPI-Reviewer incorporated more adaptable functionality that enabled more complex coding to be conducted. EPPI-Reviewer cluster formatting also provided additional information regarding the main clusters in the studies leading to a better oversight of the topics contained within the studies.

Discussions were held between the team members to identify criteria that needed clarifying at the full-text stage and to improve consistency within the results. For example, Denmark, Sweden and Germany have coastlines on the Baltic Sea but also have coastlines bordering the Skaggerak region and/or the North Sea. Team members were then assigned tasks to address the issues identified using EPPI’s search facility to find the relevant articles. For example, the articles concerning the aforementioned countries were found and the area of research identified to clarify whether it was research in one of the Baltic Sea regions or outside that area. Another example was the articles that dealt with ancient civilisations, again these were identified using the search function and excluded. A consistency check was carried out at stage 1 full text screening where we used the EPPI-Reviewer comparison mode to independently code the 110 articles (6%) by two reviewers. This consistency check was conducted in two rounds to allow discussions between team members. Inclusion/exclusion agreement was between 51% and 67.5% in the two rounds. Agreements on exclusion/inclusion was high but not necessarily on which exclusion criteria. The articles were of a diverse range where some were of a specialist, technical nature outside the scope of one of the reviewers, these articles were therefore referred for a second opinion. Referrals and disagreements were reconciled through discussion between the two team members or by further discussion with the wider team until agreement was reached. At stage 2 full-text screening, the team members agreed that all articles that explicitly mentioned “ecosystem services” or “ecosystem functions and services” in the 460 articles would be included for data extraction (n = 67).

The detailed screening process and results are depicted in Fig. 1.

### Study validity assessment

In accordance with our systematic map protocol, we have not done any critical appraisal of individual studies to assess the reliability of the evidence base. Whereas studies were excluded due to their lack of engagement with health and well-being, this was based on whether the details of the health and well-being topic were mentioned, not on the quality of the details included. The aim of this systematic map is to present a complete overview of available scientific literature.

### Data coding and strategy

Data coding we followed our coding strategy published in the protocol [8]. In addition to bibliographic information, we coded the following information:

- Study location: sea regions, coded according to the sub-basins defined by HELCOM [41].
- Research methods (e.g. study designs)
- Ecosystem services concept(s) used and sub-categories for coastal and marine ecosystem services, Table 3. The criteria used was based on Liquete et al. [45], a review article of ecosystem service categories close to our topic of ecosystem services related to the Baltic Sea.
- Health and well-being categories, after McKinnon et al. [11] which outlines domains and definitions of human well-being categories from nature conservation interventions. We used their 10 HWB outcome domains as a basis for coding, but in the results section, we further elaborate on their content empirically based on our topics' specifics.
- Mentioning of relevant policies, e.g. BSAP [4], or MSFD [3].

**Table 3:** The categories used to code coastal and marine ecosystem services (ecosystem services list based on Liquete et al. [45])

Provisioning ES	Biotic materials (raw materials; fibre; ornamental, genetic resources; biochemicals)
	Food provision (marine & terrestrial animals and plants)
	Water storage and provision
	Other provisioning ES
Regulating ES	Air quality regulation
	Biological regulation (pest and disease regulation)
	Climate and weather regulation
	Coastal protection (nat. hazard regulation & moderation of extreme events; water regulation; erosion regulation)
	Life-cycle maintenance (gene pool & genetic diversity protect.; habitat protect.; pollination; maintenance of habitats for migratory species)
	Sea nourishment
	Water purification
	Other regulating ES
Cultural ES	Cognitive effects (e.g. inspiration for culture, art & design; knowledge systems, educational values)
	Recreation, ecotourism, community activities
	Symbolic and aesthetic values (spiritual, religious, cultural, aesthetic values and experience; cultural heritage; sense of place, identity)
	Other cultural ES

Due to time constraints the data coding was shared between reviewers as indicated. FN and VL extracted all bibliographic metadata and JS extracted data for the other codes with help from MS & VL through email discussions. Independent data coding was not conducted, however TK and VL cross-checked the data after coding. Prior to the data coding the coding strategy was discussed and any uncertainties encountered by the reviewers was discussed with the rest of the team to resolve the problem.

### Cluster analysis

EPPI-Reviewer Cluster analysis software was also used in the analysis of the studies selected. The software uses text mining to assist in searching for common keywords within the articles uploaded to the EPPI-Reviewer website [46]. The articles are then automatically clustered by keyword, enabling common topics mentioned in the texts to be identified automatically. The algorithm, however, also returned topics using ambiguous words such as “load”, “other topics”, but generally the cluster analysis gave an overall insight of the topics contained within the articles before in-depth line by line coding analysis was carried out.

### Data mapping method

Data were exported from EPPI-reviewer as an excel file. Histograms were used to represent data on the bibliographic details and cluster analysis and heatmap matrixes were used to represent the data for the synthesis of ecosystem services systematic and health and well-being map findings [11, 21].

## Review findings

Our results indicated that 460 articles were focussed to some extent on the human health and/or well-being when exposed to the Baltic Sea. Often these articles were in medical/public health journals, for example, articles that discussed certain cancers (n = 36) as a consequence of exposure to the Baltic Sea ecosystem. There was a particular focus in some articles on the fishing communities of Sweden and Finland to examine the impacts from eating Baltic Sea fatty fish [16, 47–52]. Whilst these articles dealt with impacts of the Baltic Sea on human health and well-being, they did not mention the ecosystem services concept. In addition, some articles dealt with the potential of the Baltic Sea to provide important contributions to the bioeconomy through the provision of biologically active compounds with potential cancer cure properties (for example [53–55]). Again, these articles did not mention the ecosystem services. 67 articles, however, specifically did mention ecosystem services and these are the focus of this article. The database of these 67 studies is shown in Additional file 5.

### Bibliographic details

**Fig. 2:**
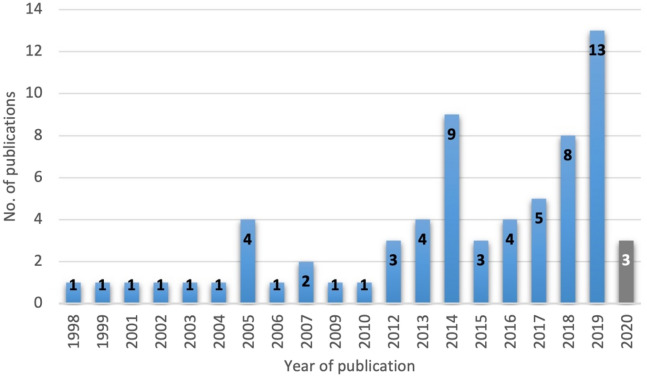
Publication years of the included full-text references (n = 67), situation at the end of March 2020. These were primarily journal articles, but also included some dissertations (see Additional file 6 details on article types)

The number of articles that report health and well-being outcomes on populations affected by the Baltic Sea ecosystem services has grown during the last two decades, although this has not always been a steady rise (Fig. 2). The distribution shows a few spikes that was be due to the planning cycles of the implementation of the MSFD, as research funding was made available to facilitate the implementation of the MSFD. The MSFD follows a six-year cycle, with Member States assessing, for example, the current status and its economic and social impact in 2012 and 2018.

**Fig. 3:**
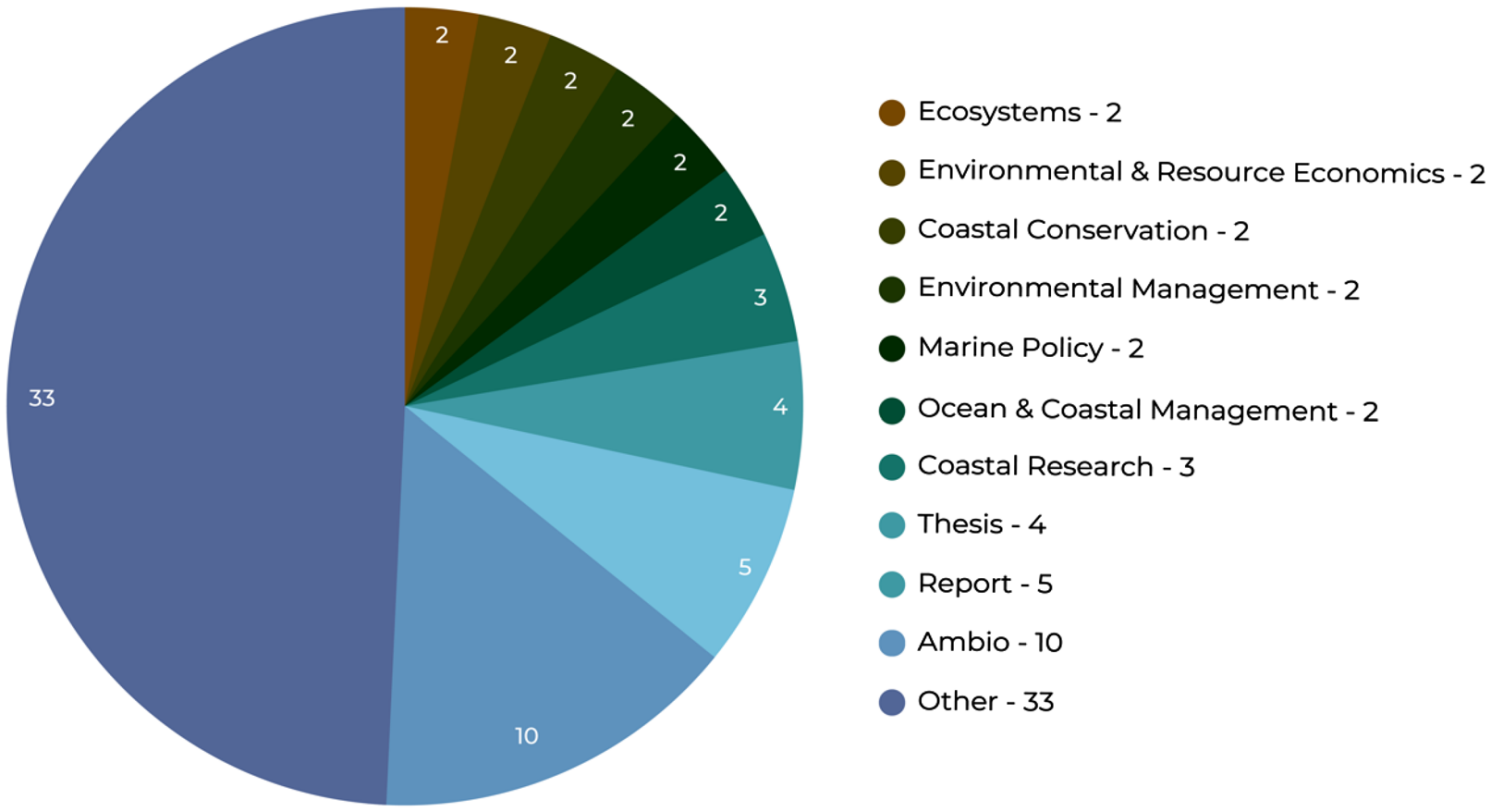
Publications by source type (other is defined as articles which were only published in one publication source)

Most of the references included were articles in journals (n = 56). The journals were from a diverse range of disciplines (see Fig. 3): 10 articles were published in ‘Ambio’ and another three in ‘Journal of Coastal Research’, the rest in various other journals (See Additional file 6 for full list).

Apart from Ambio, this figure shows that the articles are included in a variety of journals, however they are mainly environmental/ecosystem journals with a particular focus on the coastal and marine systems. Medical or public health journals do not feature in this list, which demonstrates a lack of cross-over between disciplines.

Ambio is a journal of the Royal Swedish Academy of Sciences with a focus on exploring the link of humans with their environment. The Journal of Coastal Research is produced by the Coastal Education & Research Foundation (CERF), a non-profit scientific organisation which aims to advance coastal research.

### Study dimensions, methodological trends, and geographical coverage

The codes in this section are derived from the line-by-line coding conducted in EPPI-Reviewer. The codes applied are not mutually exclusive and articles may be assigned more than one code. Out of 67 full-text reviewed articles one did not include social aspects [56] and two did not consider economic aspects [57, 58]. Fifteen articles did not consider the biophysical aspects of the Baltic Sea, they focussed on management of the Baltic Sea, its aesthetic qualities and the nutrients in pollution.

**Fig. 4:**
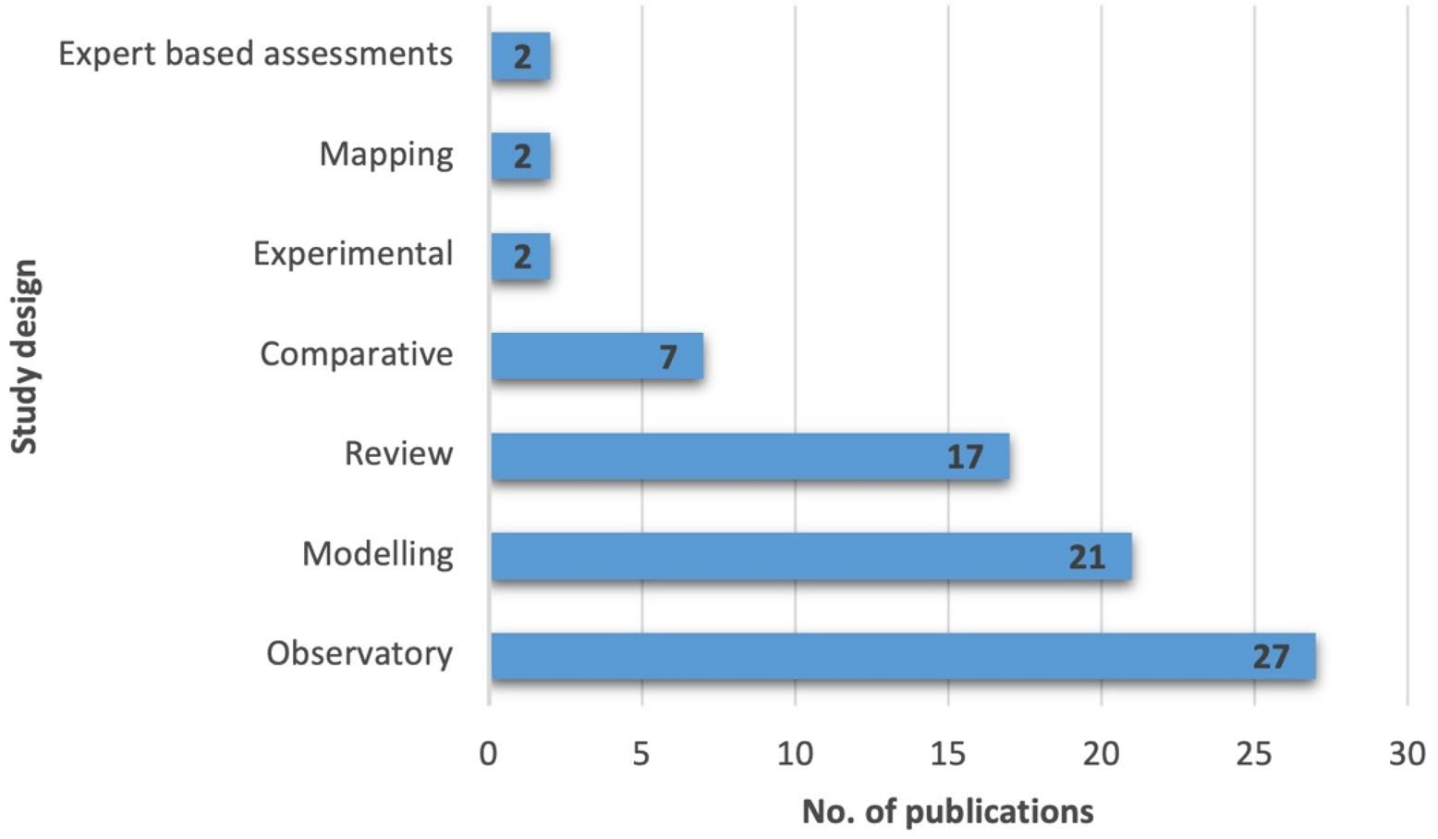
Study design formats of the included full-text references (n = 67). Some articles were double-coded. Observatory refers to case studies where populations or areas were observed. Review refers to articles that took a selection of papers to examine a topic. Modelling refers to the theoretical development of concepts. Comparative refers to articles with multiple site observations. Mapping refers to either the mapping of ecosystem services or quantifying of a component of an area, for example nitrogen retention of a wetland. Experimental refers to a study where parameters are tested, for example choice experiment

Study designs were largely observatory (Fig. 4), particularly in valuation articles that assessed how willing people were to pay to offset damage to ecosystems, or the value they placed on an amenity (n = 16). Modelling studies also reflected a focus on how to value or measure the ecosystem services (n = 16).

**Fig. 5:**
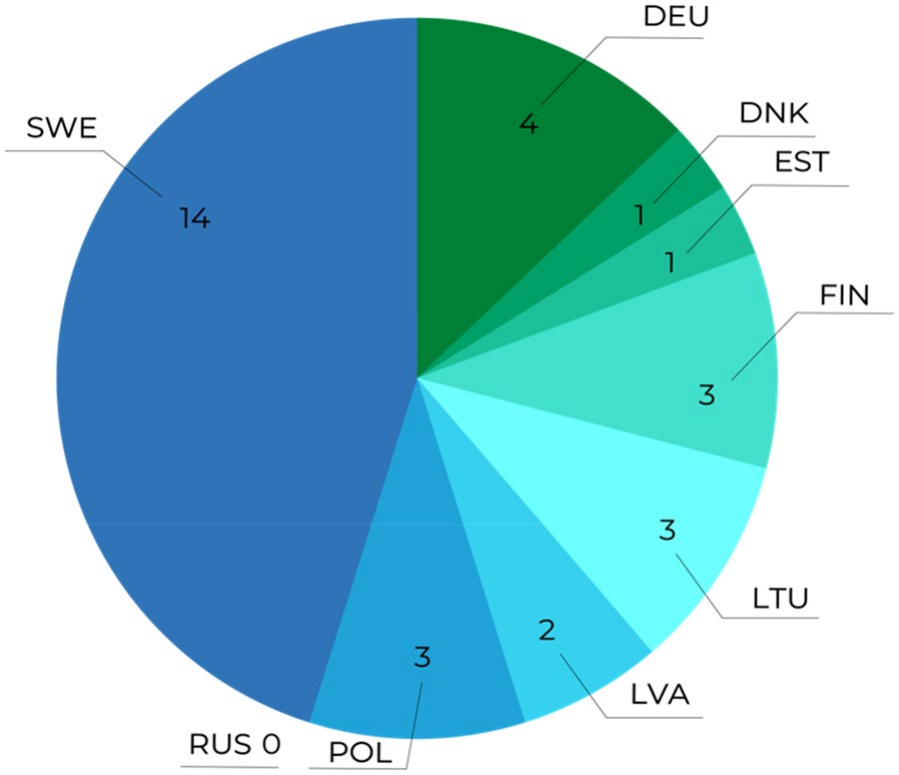
Articles by country: Research carried out in a single country and labelled according to ISO country codes. Countries include all Baltic Sea Countries: Sweden (SWE), Germany (DEU), Denmark (DNK), Estonia (EST), Finland (FIN), Lithuania (LTU), Latvia (LVA), Poland (POL) and Russian Federation (RUS)

Most of the articles were focussed on a single Baltic Sea country (n = 31, see Fig. 5) or covered all the Baltic Sea or all the Baltic Sea coastlines (n = 16), all other articles covered 2–4 Baltic Sea countries (n = 18) apart from two studies which included one Baltic Sea country and other non-Baltic Sea countries.

**Fig. 6:**
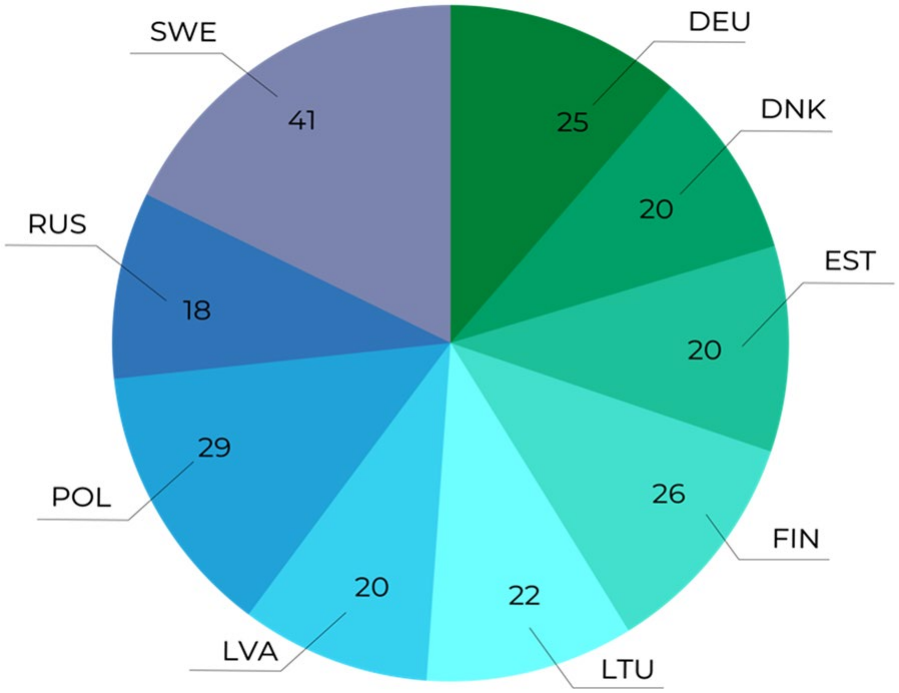
Spread of articles by country: multiple countries may occur in one article

Most articles were multi-national collaborations (n = 38) and reflected the focus of funding on collaborative investigations or the focus on the whole of the Baltic Sea basin. Figure 6 shows the distribution of countries covered by all articles. Apart from the Swedish examples, generally there was an even spread of countries investigated that reflected the focus on the whole of the Baltic Sea rather than specific sea basins in many of the articles.

**Fig. 7:**
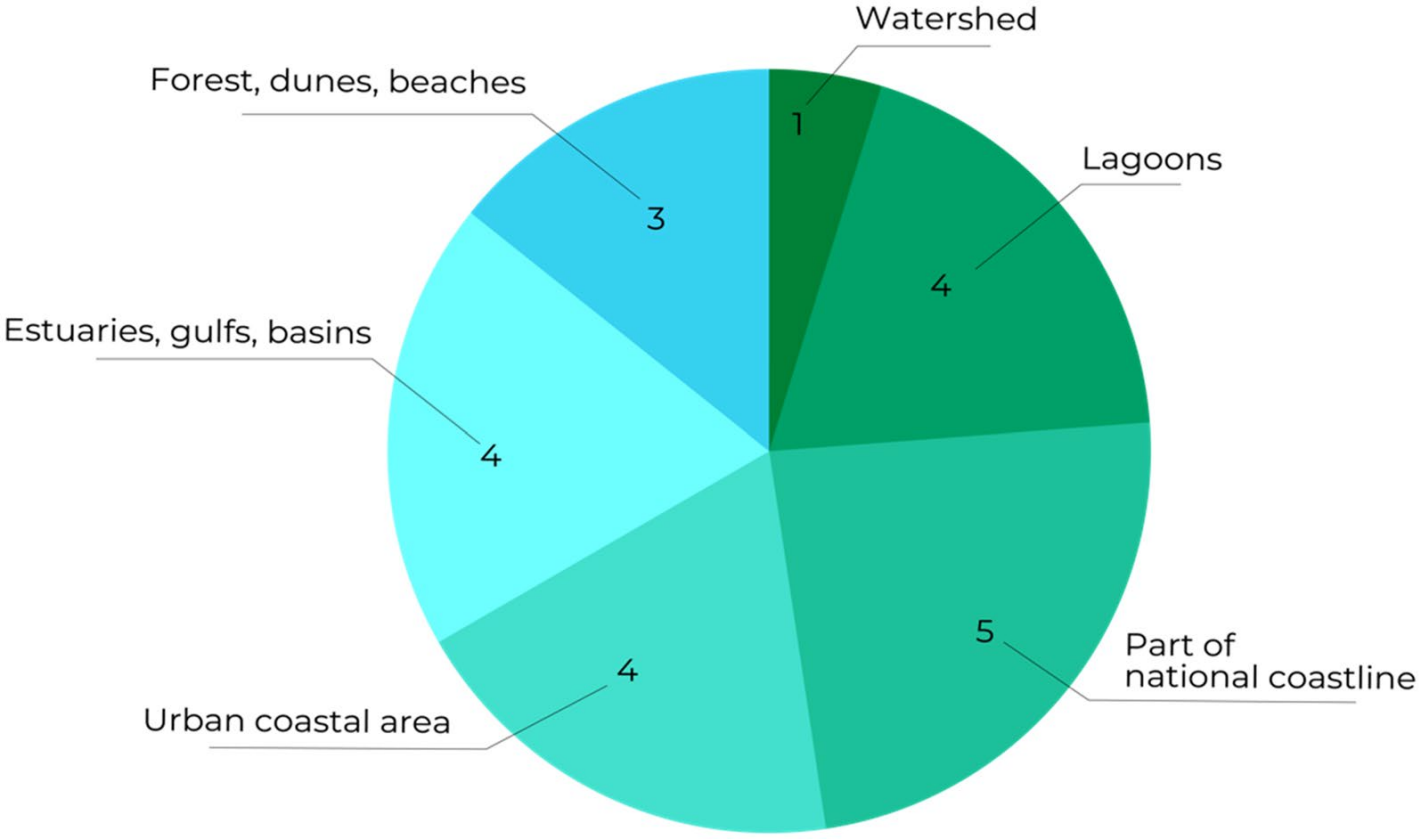
Study scale: typology of locally coded research

Twenty-one articles were coded as “local” in scale. For the purpose of this study, local was defined as referring to either a part of a national coastline or multiple well-defined sites, such as lagoons or urban areas. These sites could be based in one country (n = 14) or multiple countries (n = 7) (see Fig. 7).

Sixteen articles focussed on national coastal areas, of which 7 were based on the Swedish coastline. This reflects the influence of the SUCOZAMA programme [14], which ended in 2004 and contributed three of the seven Swedish articles that explicitly covered ecosystem services and health and well-being topics. Other articles were generated from this programme but did not explicitly mention ecosystem services and/or did not include health and well-being so were not included in the articles selected.

There was an almost equal share of the studies that reported on only marine (n = 8) and only coastal (n = 7) areas, and most (n = 52) of the articles dealt with both the marine and coastal areas.

**Fig. 8:**
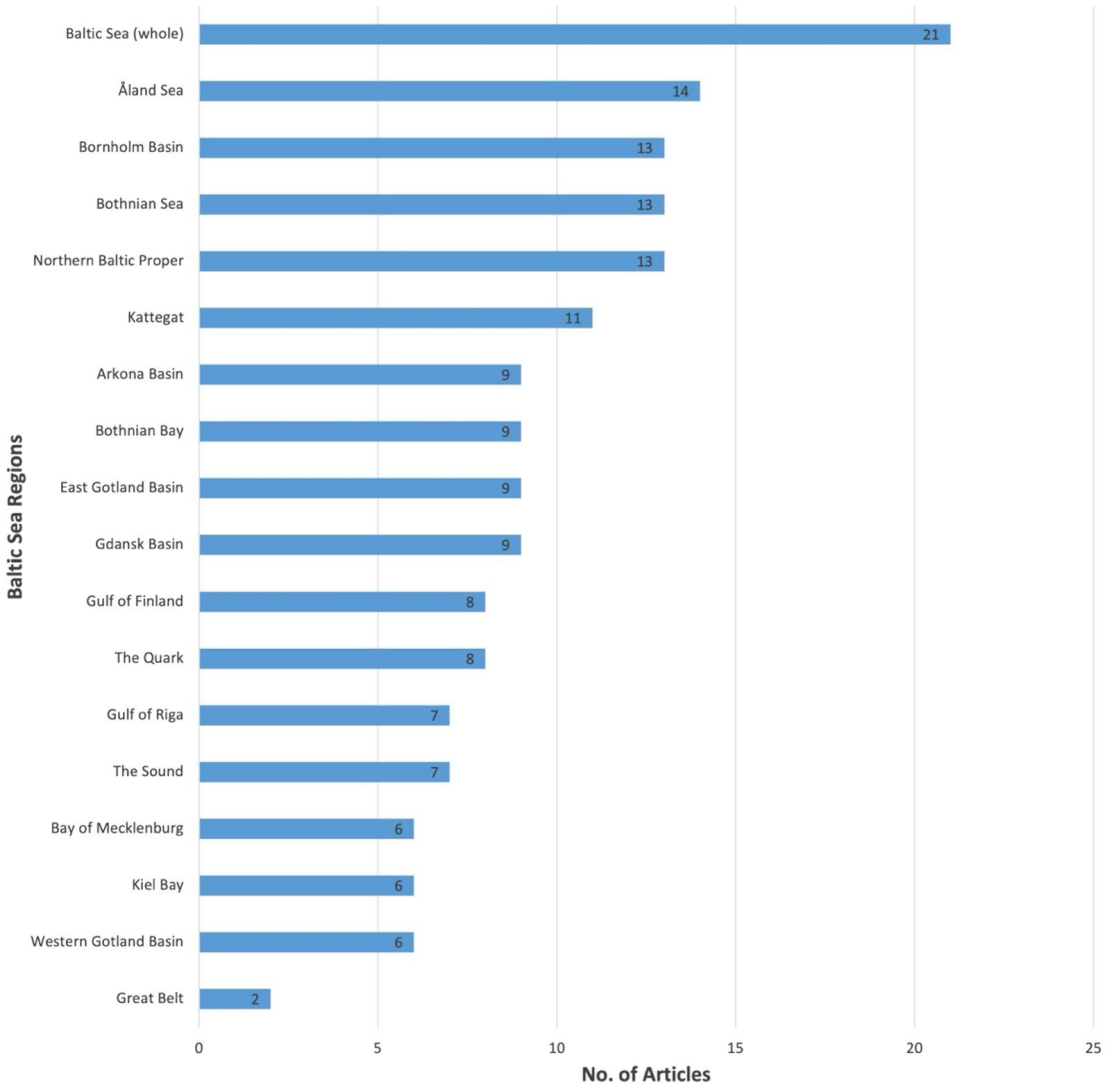
The HELCOM sea regions [41] included full-text references (n = 67). Note the codes are not exclusive and therefore one study can refer to more than one HELCOM region

As mentioned, many articles were based on studies of the Swedish coastal areas, particularly in the Stockholm area either as a single nation study (n = 13), or in conjunction with Finland (n = 2) or as part of the Baltic Sea as a whole (n = 17). The greatest number of sea basins represented, therefore, were the Åland Sea and the Northern Baltic Proper based in Swedish waters. The area represented by the Great Belt (between the island of Zealand and mainland of Jylland, Denmark) was only included in articles that covered the whole of the Baltic Sea but not studied separately (Fig. 8).

### Link to coastal and marine ecosystem services

**Fig. 9:**
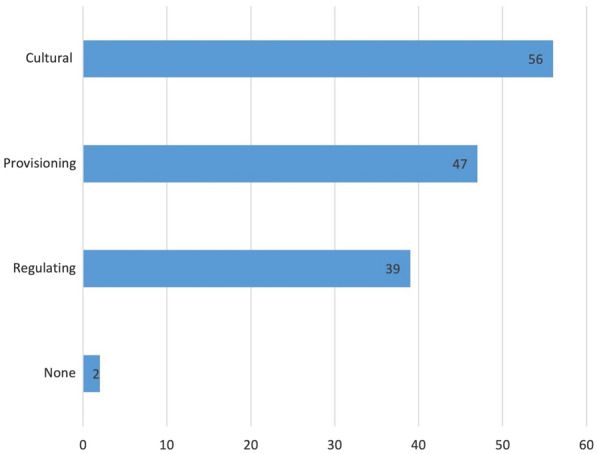
The mentioned ecosystem service sections in the articles (n = 67). Articles may have more than one ecosystem service mentioned

**Fig. 10:**
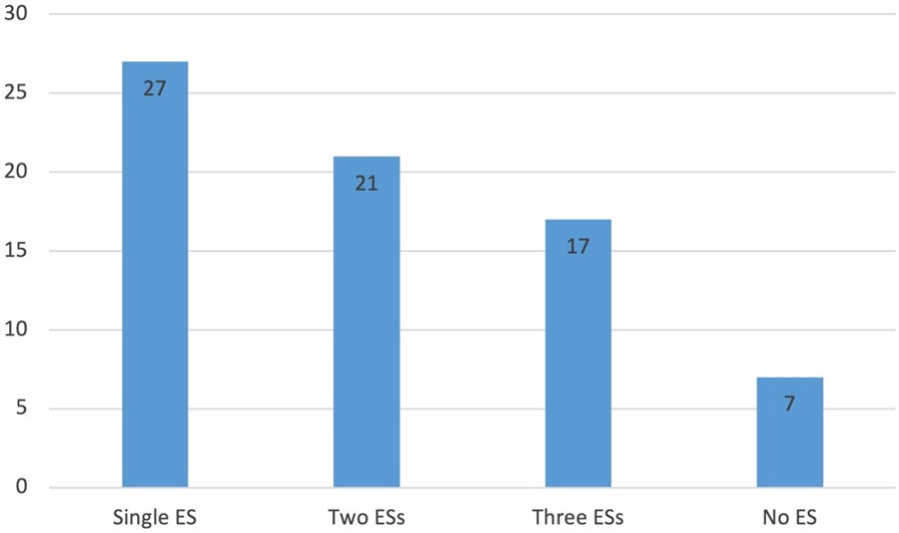
Number of different ecosystem services (ES) mentioned in the articles (n = 67). Two articles did not mention any specific ecosystem services

Figure 9 demonstrates that cultural ecosystem services were the most mentioned of ecosystem services in the articles (n = 56). Figure 10 illustrates the number of different types of ecosystem services that were covered in each article. Two articles, however, despite explicitly mentioning ecosystem services made no further link to any specific sections or services (labelled “none” in Fig. 9). The mention of the ecosystem services, however, did not mean the topic was engaged with in depth, as evidenced by the lack of content devoted to the topic.

### Outcome categories in human health and well-being

It was noted in the articles that some ecosystem services were documented as having a positive effect on health and well-being but the perceived benefits by the general public was low. For example, an increase in reeds will improve the stabilisation of the seashore, as well as filtering pollutants in the water. However, reeds are often perceived negatively by tourists due to a lack of understanding of the benefits, thus an increase in economic living standards and health but a decrease in subjective well-being [59, 60].

**Fig. 11:**
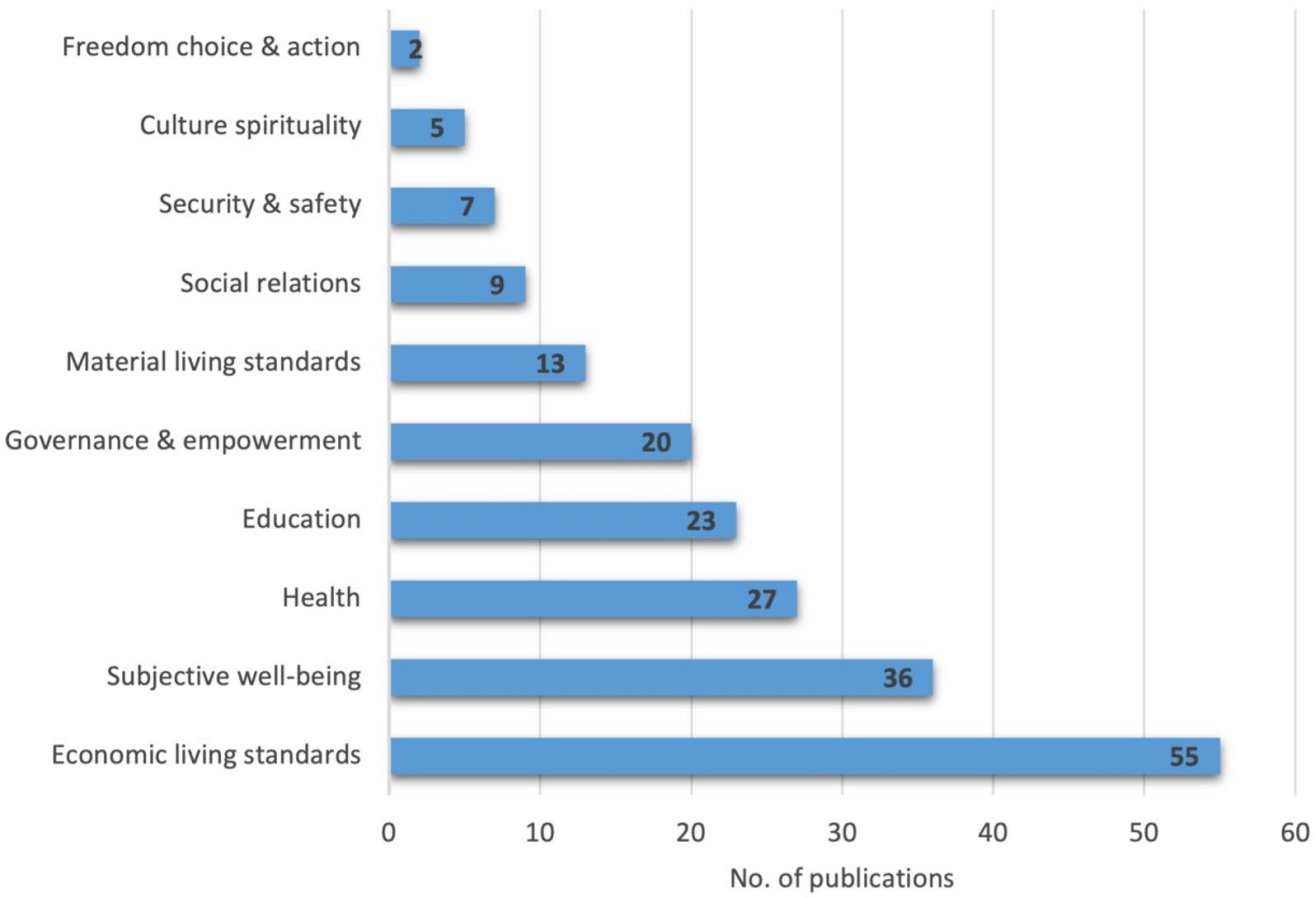
Health and well-being outcomes identified in the articles (n = 67) according to the definition of health and well-being domains in McKinnon et al. [11]. Articles may have been coded with more than one code

The results of the outcome categories in human health and well-being according to the categories as outlined in McKinnon et al. [11] are shown in Fig. 11. The three most mentioned outcomes were related to the categories of Economic living, Health, and Subjective well-being. In general, education was often mentioned in a negative context as the need for more education was often highlighted. It was stated that this was due to a lack of knowledge regarding marine ecosystems and mitigation measures that hampered conservation and planning within the Baltic Sea marine environment [61–64]. There is also a lack of knowledge regarding the socio-economic linkages to the ecosystem services [56, 65, 66]

As shown in Fig. 11, economic living standards featured in most of the selected articles, particularly linked to activities derived from tourism and fishing associated with the Baltic Sea. Subjective well-being also featured highly due to the number of valuation studies conducted, which examined the perspectives of people exposed to the Baltic Sea. Freedom and choice, however, was rarely covered but covered topics such as managed realignment of coastal areas [67] and the loss of sand from beaches [68] and the choices needed to address the issue. This aspect will be especially important due to the impact of climate change on coastal communities and therefore represents a gap in the literature.

### Reported links between ecosystem services supply and human well-being

**Fig. 12:**
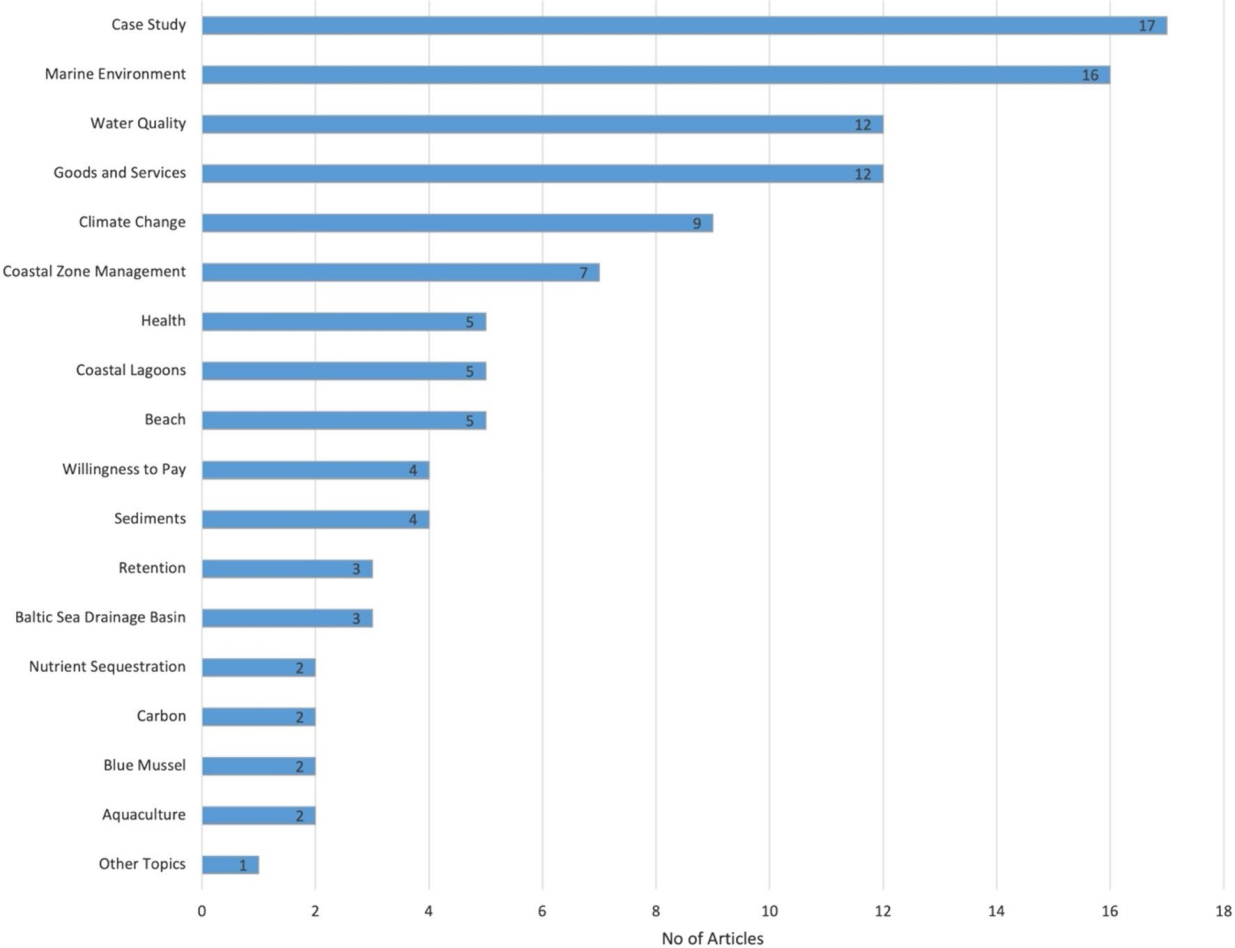
Topics identified by cluster analysis linking ecosystem services and human well-being in the mapped articles (n = 67). Articles may be coded with more than one code

The results from the cluster analysis of the 67 articles that mentioned ecosystem services are presented in Fig. 12 (full list of articles in Additional file 7). As highlighted by the recent Covid 19 pandemic, pathogens have potential catastrophic impacts on human health and well-being, however this study only found one article [57] that linked pathogens to ecosystem services in the Baltic Sea. Other zoonoses were identified (n = 13) in the 460 articles but not explicitly linked to ecosystem services. The most common connection to ecosystem services was made in coastal management or marine spatial planning articles. As this is a computer-generated analysis, the machine algorithm makes a distinction between coastal management and marine spatial planning. This highlights the varying terminology used between articles.

### Synthesis of ecosystem services systematic and health and well-being map findings

**Fig. 13:**
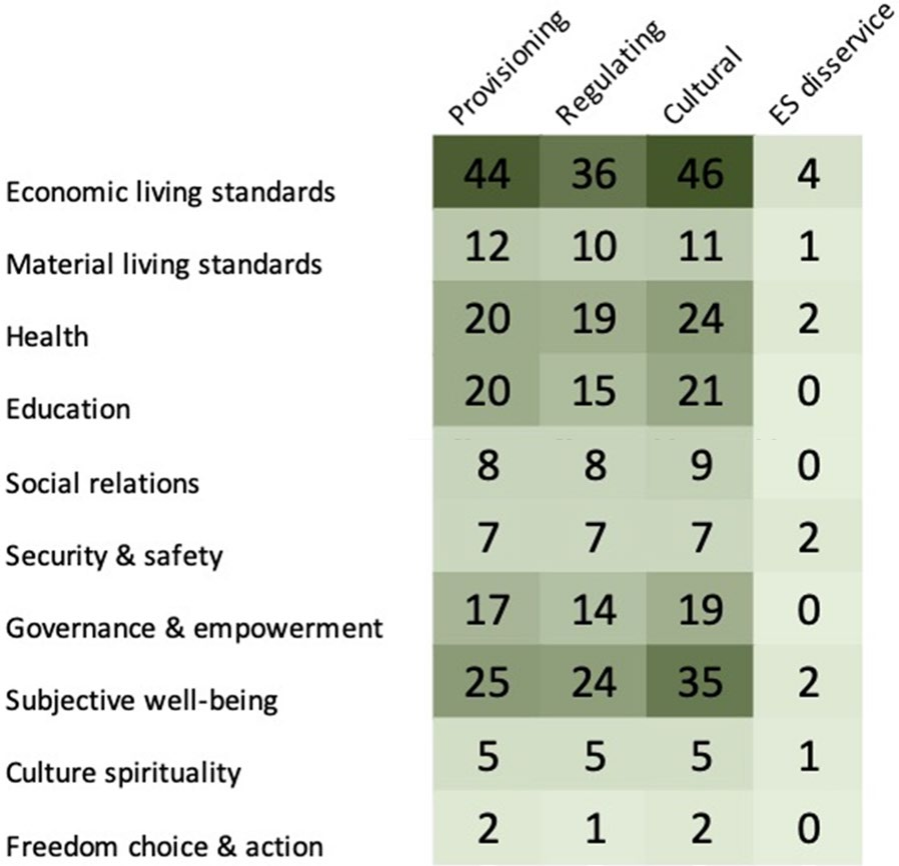
The distribution of articles between the ecosystem services of provisioning, regulating and cultural services plus Ecosystem disservices and the indicators for health and well-being. Knowledge clusters are shown in a stronger green with knowledge gaps represented by a pale green colour (see Additional file 8 for interactive map with references created with eppi-mapper software [69])

The ecosystem services coding was cross tabulated with the health and well-being domains coding to produce heatmaps. The heatmap matrixes show the distribution of articles and the linkages between ecosystem services and human well-being. Heat map matrixes are a useful visual method of representing the distribution of the evidence on a topic and highlights the gaps in knowledge with a lighter colour. Figure 13 shows there is an even spread of articles across the various ecosystem services as generally they were all mentioned in the articles examined. However, the greatest focus was on Economic living standards with Freedom of Choice and Action the least studied.

There was a discrepancy between the way that articles referred to ecosystem services. Some articles mentioned the term “ecosystem services” but did not specifically mention what kind of services were provided, this had to be deduced from the topics covered. Some specifically mentioned the terms provisioning ecosystem services, regulating ecosystem services or cultural ecosystem services. The first column specifically refers to articles that mentioned these services.

**Fig. 14:**
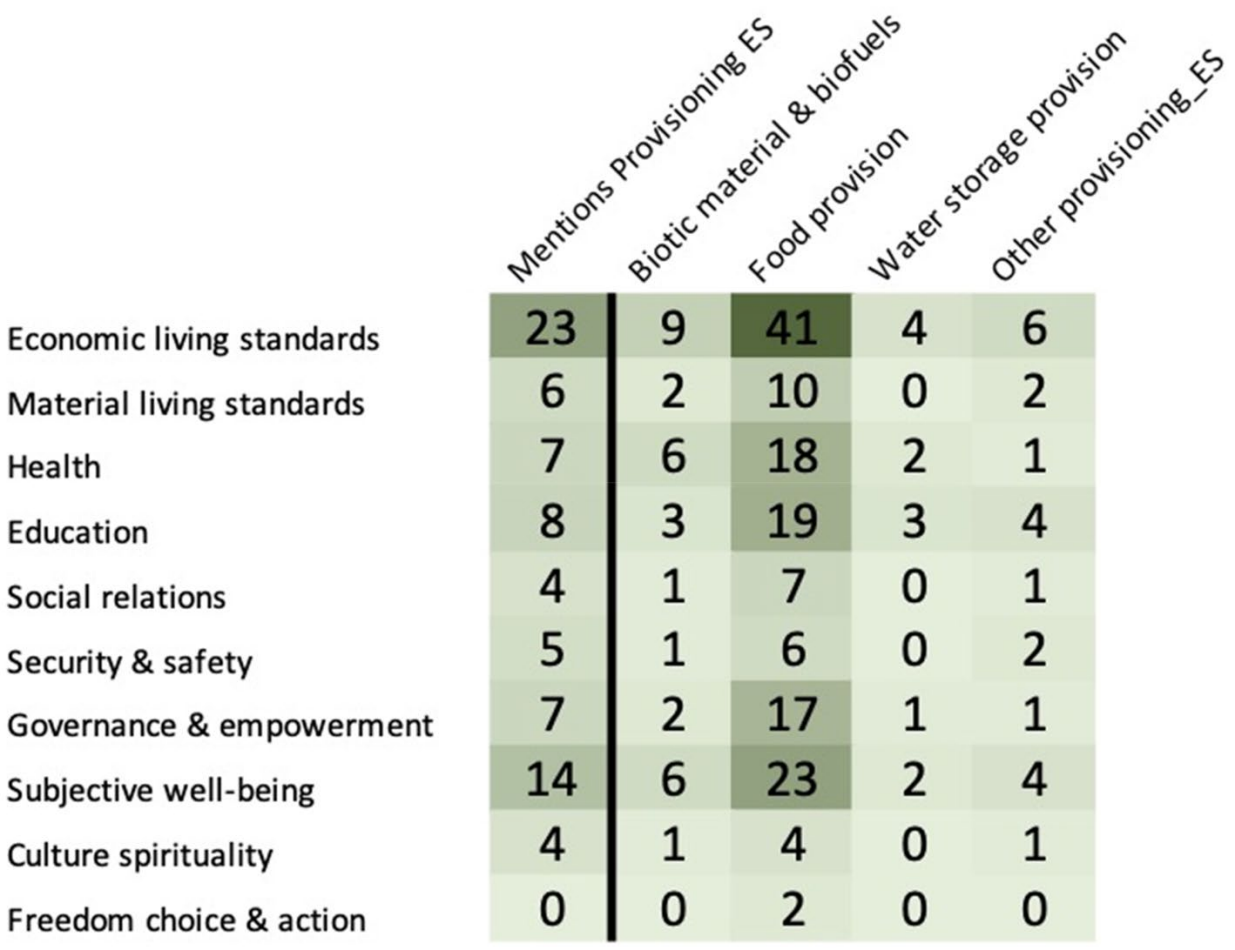
Distribution of articles between Provisioning ecosystem services and health and well-being. The first column shows the distribution of articles that specifically mentioned provisioning services and the health and well-being indicators. The four columns together show the distribution of articles by the different provisioning ecosystem services as outlined in Table 3 and health and well-being indicators in Table 1 (see Additional file 9 for interactive map with references created with eppi-mapper software [69])

Figure 14 demonstrates that the main provisioning service mentioned is the provision of food. All other provisioning ecosystem services are neglected aspects of the provisioning service. Of these articles 27 mentioned fishing, either commercial or recreational, 4 mentioned mussels and 6 mentioned shellfish.

**Fig. 15:**
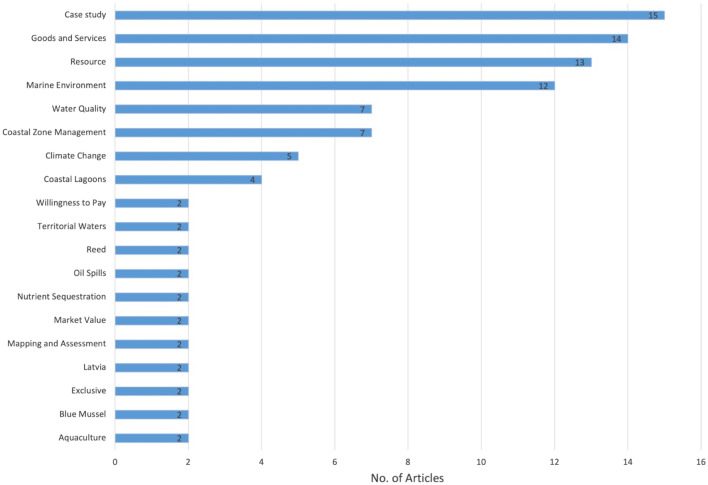
Cluster analysis of provisioning ecosystem services. Articles may be coded with more than one code

Cluster analysis was also applied to articles coded with provisioning ecosystem services, it was found that articles on food provisioning included topics such as aquaculture (n = 2) and blue mussels (n = 2) but most focussed on the management of the Baltic Sea and its coastlines, which encompasses management of fish stocks or the fishing industry to minimise impacts or they focussed on topics that have a potential impact on provisioning such as climate change, water quality, oil spills and so on (see Fig. 15 and Additional file 10).

**Fig. 16:**
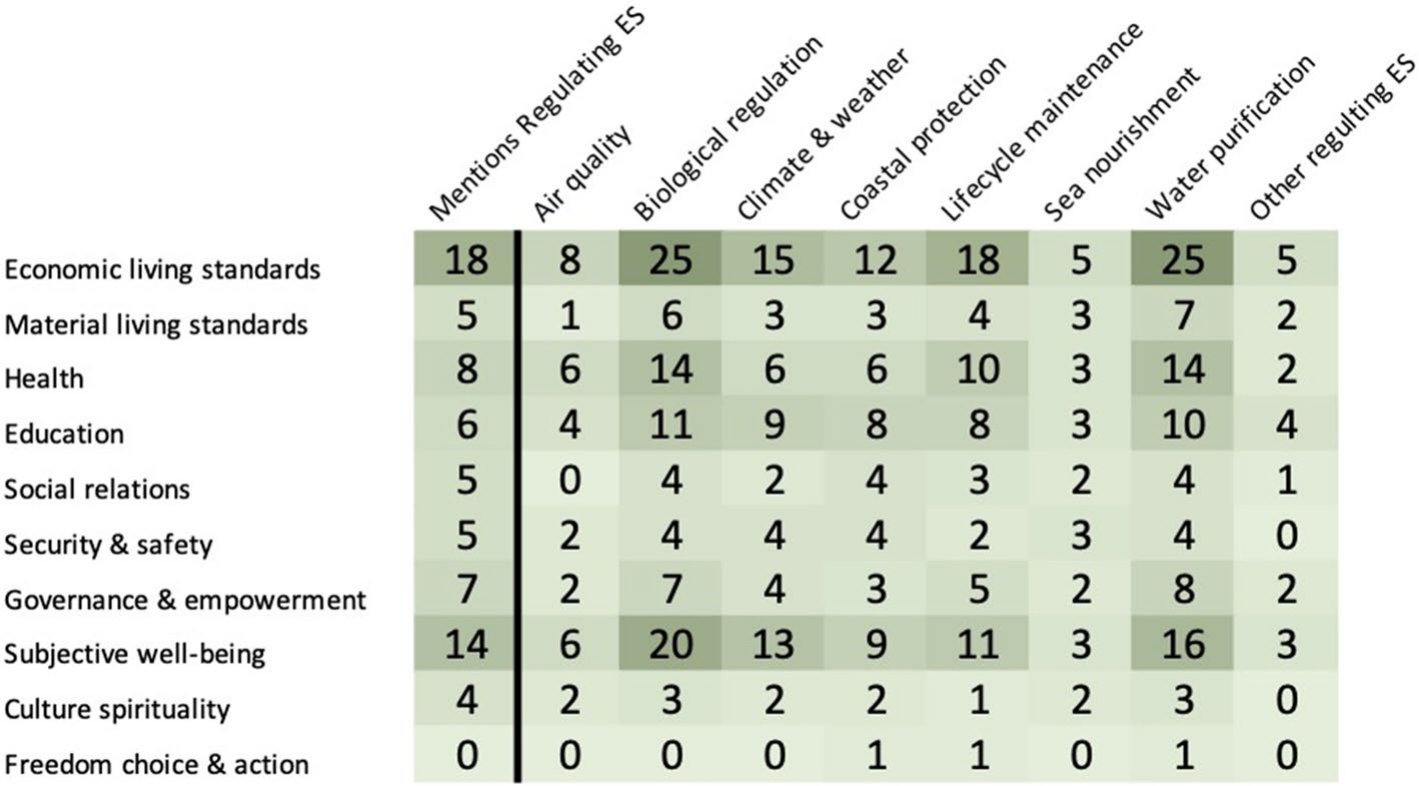
Distribution of articles between Regulating Ecosystem Services and Health and Well-being. The first column shows the distribution of articles that covered the various health and well-being indicators and those articles that specifically mentioned regulating ecosystem services. The following columns together show the distribution of articles by the different regulating ecosystem services as outlined in Table 3 and health and well-being indicators in Table 1. (see Additional file 11 for interactive map with references created with eppi-mapper software [69])

Research on regulating ecosystem services focussed mainly on biological regulation [7], the main topics covering eutrophication and wetland reed systems. The most common health and well-being indicator studied was economic living standards (Fig. 16).

**Fig. 17:**
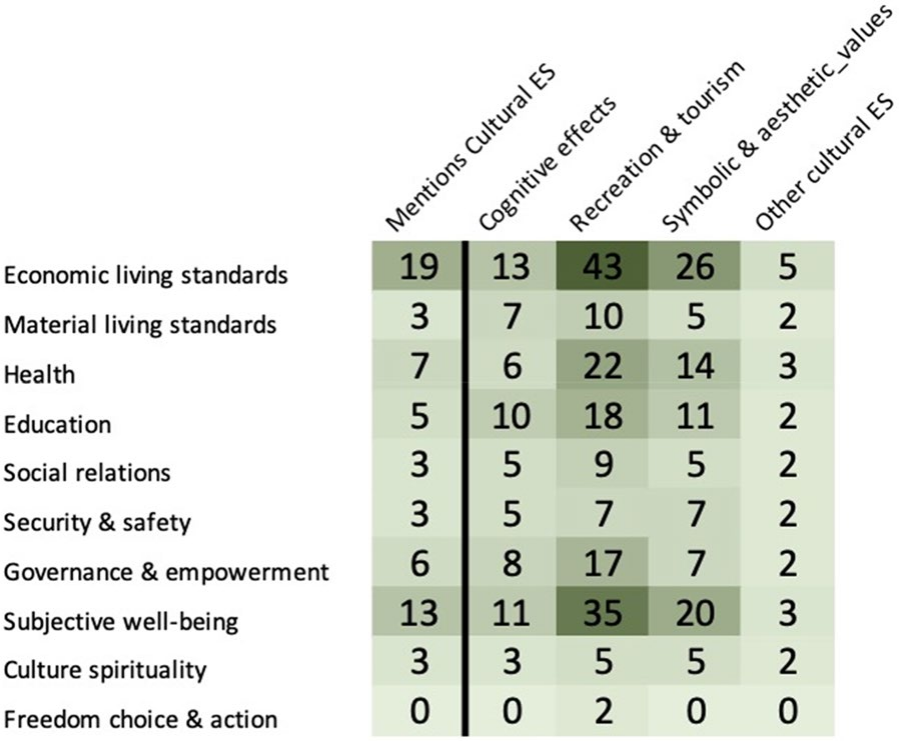
Distribution of articles between Cultural Ecosystem Services and Health and Well-being. The first column shows the distribution of articles that covered the various health and well-being indicators and those articles that specifically mentioned cultural ecosystem services. The following four columns show the distribution of articles by the different cultural ecosystem services as outlined in Table 3 and health and well-being indicators in Table 1 (see Additional file 12 for interactive map with references created with eppi-mapper software [69])

Cultural ecosystem services tended to focus on Recreation and Tourism, which has obvious benefits for economic well-being and subjective well-being. Only two articles examined aspects of Freedom of Choice and Action and these were both valuation methods assessing the choices people would be willing to take to see less eutrophication in the Baltic Sea environment (Fig. 17).

### Policy relevance

**Fig. 18:**
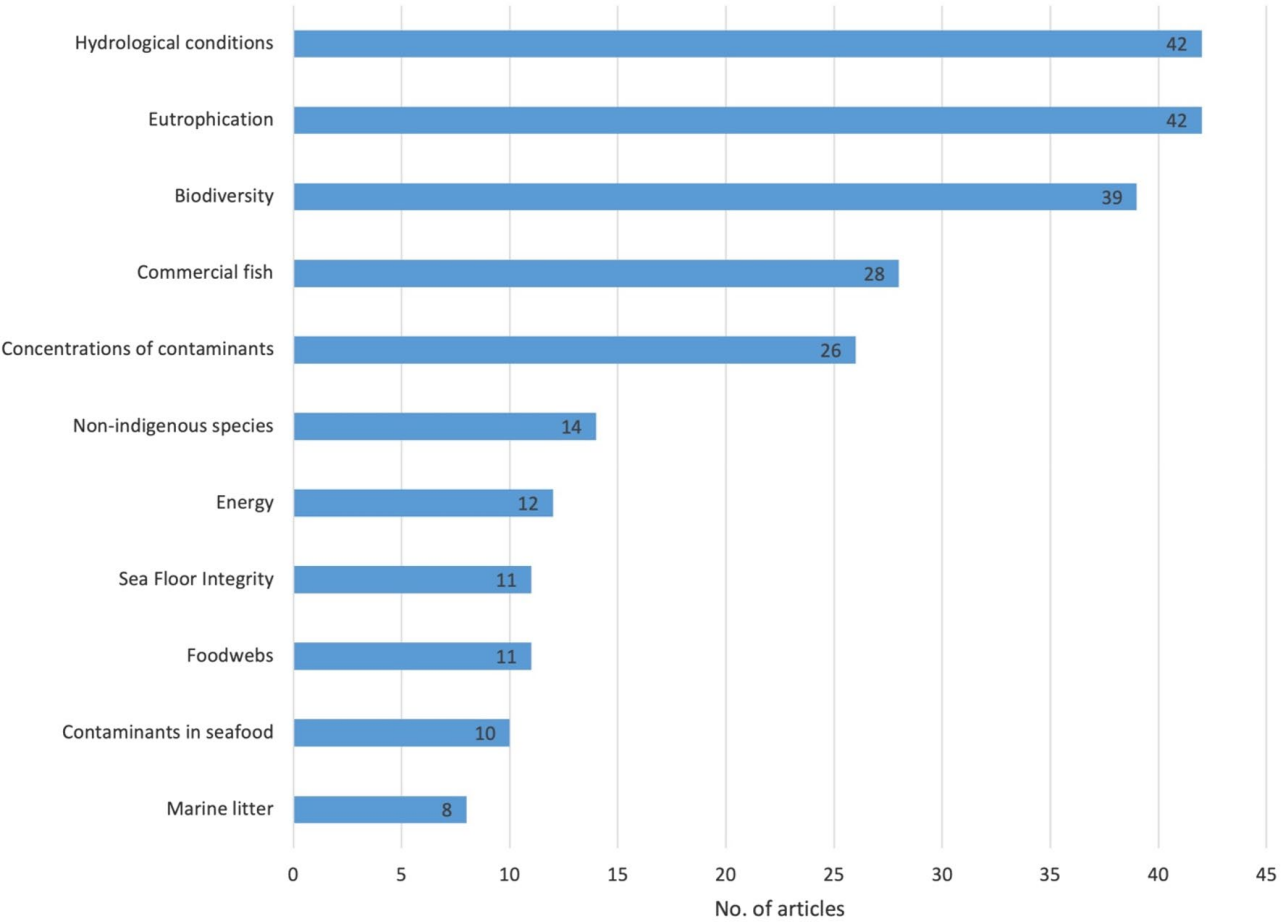
Number of articles with the descriptors of good environmental status (n = 67)

To establish objectives for the achievement of Good Environmental Status (GES) within the implementation of the MSFD, 11 GES descriptors have been selected to aid the assessment of the environmental status of marine waters [70] (Fig. 18). Only three articles did not mention any reference to a topic covered by a GES descriptor. The most cited descriptors were “Permanent alteration of hydrographical conditions does not adversely affect the ecosystem, Descriptor 7”, “Eutrophication is minimised, Descriptor 5”, and “Biodiversity is maintained, Descriptor 1”. Only ten articles dealt with contaminants in seafood and eight articles dealt with marine litter.

The BSAP was explicitly mentioned in 18 articles. Articles, however, covered the topics mentioned in the BSAP goals, eutrophication, hazardous substances, maritime activities biodiversity, without explicitly mentioning the BSAP itself. For example, the most commonly mentioned BSAP goal was reduced eutrophication which occurred in 18 articles, of which 11 also mentioned the BSAP. Whilst the majority of articles did not mention any of topics related to the BSAP goals 15 mentioned one of the topics related to the goals and 4 mentioned more than one topic related to the goals.

### Identified gaps in the evidence base

As the interventions with stakeholder participants indicated, the systematic map demonstrates that the evidence linking health and well-being indicators to ecosystem services is low: only 67 out of 460 articles that mentioned health and well-being indicators linked to the Baltic Sea specifically mentioned ecosystem services. In addition, the evidence base generally lacked depth in either the ecosystem services concept and/or the health and well-being concepts. Indeed, many articles that engaged with the ecosystem services context did not elaborate on the factors contributing to consequences on health and well-being resulting from the ecosystem services provided. Primarily those articles dealing with cultural ecosystem services expanded on these concepts to some extent. Culture and spirituality connected to food provision is low and yet food provided from the sea is rich in cultural significance based on its long history [71].

Generally, these results demonstrate that the links made in the literature between ecosystem services provided by the Baltic Sea and health and well-being are low despite the development of the ecosystem services concept over several decades. In order for policy advisors to be provided with the relevant information and a better understanding of the connections between the Baltic Sea ecosystem services and health for the development of the Baltic Sea Action Plan in the next cycle there is a need for additional interpretation of the 460 articles that mentioned health and well-being indicators, which is beyond the scope of a systematic map.

Human influence interferes with the potential ecosystem service supply, as they form part of the feedback loops that impact ecosystems [23]. Therefore, humans need to be taken into account in the management of the Baltic Sea, not just as the source of harm, but also as part of the system that is impacted. The lack of scientifically proven linkages between health/well-being and marine ecosystem services, hampers policymaking and management of the issues. For example, there were many articles that detailed the presence of persistent organic compounds in food and the impacts on human health, but these did not provide links to how persistent organic compounds are circulated and distributed through the ecosystem from both natural and anthropogenic origins to end up in food to the extent where it impacts human health. For example, studies by Axmon on female fertility and fish consumption [72–75], Rigell-Hydbom on fertility in Swedish fishermen [76] and Turunen on the incidence of cancer associated with high fish consumption [52]. The issues of contamination of fish by harmful substances tend to be dealt with at the point-of-sale level and not linked to the issues of the harmful substances in the ecosystem, except for perhaps a brief mention. Linking these concepts would lead to an impetus to solve these complex issues, where contaminants harm the environment and potentially impact human health and well-being.

Our results clearly showed that joint research between different disciplines is needed to demonstrate the potential that the Baltic Sea ecosystem services provides to health and well-being. However, the information needed to demonstrate the potential that the Baltic Sea ecosystem services provides to the health and well-being of the populations that are exposed to their environment is largely missing and further analysis is required to link these to ecosystem services or disservices provided. Furthermore, enhancing the science-policy-society interaction is crucial to advance the knowledge-base of the stakeholders to integrate ecosystem services and their connection to human health and well-being in marine management.

In this study, we focussed on the 67 articles that specifically mentioned ecosystem services to answer our original question, “What linkages have been researched between Baltic Sea ecosystem services and the positive and negative impacts to human health and well-being?” in accordance with the published protocol [8]. This demonstrated that despite the inclusion of ecosystem services into the MSFD, it has not been integrated into the research in the Baltic Sea and its connection to human populations. A lack of information connecting these two topics hamper the availability of scientific evidence to inform policymakers. In this selection of articles, the lowest number of hits referred to contaminants with sea food and marine litter and yet overall, this constituted a significant number of articles that engaged with human health in the larger group of 460 articles. This suggests that while the link between contaminants and pollution to human health and well-being is well researched, the link has not been made to the ecosystem services provided. This highlights the need to integrate the articles that describe health and well-being influenced by the Baltic Sea environment into the ecosystem services they provide, thus strengthening the argument that conservation of the ecosystems is beneficial to human health and well-being too.

As Armoškaite et al. [26] and Blythe et al. [12] recently stated, there are plenty of theoretical models regarding the ecosystem services supply (models included scenarios, socio-economic, bio-economic, biophysical/chemical, anthropogenic pressure, planning and emergency response models) but there is a lack of practical applications to demonstrate the models work. We also found this to be the case as our systematic map highlights that much of the empirical research is falling behind theoretical developments in the field. Observations were mainly limited to socio-economic valuations linked to cultural ecosystem services, only one engaged with health-related issues [58]. As Blythe et al. [12] found, most articles highlighted the connection of health and well-being to ecosystem services but did not sufficiently expand on those relationships. Ecosystem disservices were rarely mentioned and considering the potential impacts on human health and well-being this is a significant gap in the literature. This may reflect the more recent introduction of the term, or as Dunn [29] describes a missed opportunity to protect ecosystems in order to mitigate harmful effects on health and well-being of the human population.

### Limitations in the process of systematic mapping

#### Limitations of searching

Keyword searches are an important aspect of academic searches but as mentioned, there were problems using this feature in Web of Science, as their KeyWords plus search returned too many irrelevant results. This feature has since been amended in Web of Science and it is now possible to search with author keywords only and ignore the KeyWords plus feature. This also had repercussions on searches in other databases to ensure comparability.

To understand the inter-relationships of the ecosystem services and their impacts on human health and well-being, we need inter-disciplinary investigations and literature syntheses. This will necessitate the clarification of terms by a variety of experts to link the ecosystem services terminology to the different health and well-being aspects. For example, the use of the word “fisheries” can denote a fishing ground, or the occupation, or industry related to catching fish. The word “toxic” or “harmful” is often used without specifying to whom or what the substance is toxic or harmful. Some toxicities are species specific. This required checking with the European Chemical Agency to ascertain which chemicals were harmful to humans [77]. In the meantime, it is important for literature syntheses to recognise the multiplicity of terminology used and issues around the use of common words that may require extensive use of the Boolean operator “NEAR” to be used to ensure the relevant literature is included. This fluidity of the use of terms also presented difficulties in interpretation of the articles from time to time, especially when using cluster analysis. Fisheries for example can refer to the fishing industry and the area where fishing takes place and therefore requires careful reading of the text to ensure the correct interpretation of the term has been used.

National databases were used where the BONUS ROSEMARIE team members expected to find information on ecosystem services relevant to the project teams countries in English. These databases took a great deal of time to search and it is suspected that there was a great deal of overlap with the other search engines. From the information science point of view, it would be very interesting to do an overlap-analysis. This analysis would uncover where the references included in the final stage of the assessment were found. It would reveal which references came from outside the scientific reference databases (which were searched first) in other words, whether searching other databases/archives was a waste of time and resources.

#### Limitations in coding and synthesis

As mentioned, ecosystem services and disservices cover a wide range of topics requiring inter-disciplinary research teams. Our team had a wide range of experience, such as ecosystem services, pharmacology, chemistry, sociology, environmental management, marine policies and conservation, but it is not possible to cover all disciplines in depth with the resources available, therefore, some articles may have been coded differently to the way that experts in the specific fields would code. This is a particular problem with the health and well-being aspects that covers topics in sociology, economics, health and so on.

Of particular note is an issue regarding access to subscription databases and the subsequent access to publications. Our multi-country team meant we had a wider access to the databases, but it should be noted that test searches showed that access varied depending on the site from which they were accessed. In addition, this means that important information for policy advisors and decision-makers on health and well-being connected to ecosystem services may not be available to them. Additional research is therefore needed into the site specificity of systematic research. It also means that we may be missing some articles that were not accessible to us. Policy advisors and decision-makers need access to the most up-to-date information either directly or through academic collaboration to reach the best decisions to manage ecosystems for the health and well-being of people and the environment, as such this requires access to all research on the topic.

### Specific coding issues

One major coding issue was related to the assignment of correct geographic locations of the articles. Sea basin labels were generally taken from the Helsinki convention; however, these labels were not consistently used in the articles. In addition, difficulties were encountered with some of the official names, for example “The Sound”, also called Øresund in Danish, which lies between Sweden and Denmark, returned too many irrelevant articles. Similarly, “The Quark” returned articles related to physics, or the alternative Kvarken was used. Some articles used the term “Baltic Sea” but this did not refer to the Baltic Sea as a whole but on a specific basin; coding in these cases was based on the maps included in the articles.

Other coding issues arose due to the fact that the focus of many of the articles were not on health and well-being indicators and these had to be inferred from the text. Some codes are easier to identify such as those connected with health, but others, particularly “Freedom of choice and action” are less clearly articulated in the texts and perhaps not well understood as an indicator for health and well-being. In order to ensure transparency in coding, the records for the text assigned to the specific code will be held in a repository that can be accessed on request.

### Time factor

The BONUS ROSEMARIE project had an ambitious timescale that was not apparent at first. Learning the systematic mapping process took a significant proportion of the initial stages. Stakeholder dialogue was important to the process, but stakeholders had limited availability to devote to the project, therefore opportunities had to be taken to run workshops and interviews that fitted within the timetable of other meetings that stakeholders were already attending. Time taken to get the protocol accepted whilst trying to maintain the forward momentum of searches also proved problematic. The extra time factor of checking through the various disciplines involved added extra time, despite the range of expertise on the team. Haddaway and Westgate [78] have undertaken considerable work in elaborating the processes undertaken in systematic reviews and mapping, however, it does not take account of the protocol’s publication process that can prove challenging in projects with shorter timeframes. Whether to publish the protocol or not is a pertinent question in such cases.

### Overall outcome

It should be noted that in the wider collection of articles (n = 460) there is more information regarding the impacts of the environment on human populations, such as eating fish, that shows the benefits to the diet and the disbenefits of eating contaminated fish from the Baltic Sea, but this is not explicitly linked to the ecosystem services concept. As discussed by Rivero and Villasante [13] and verified by the interviews with stakeholders, the ecosystem services concept is considered a helpful concept to demonstrate the interdependency of human populations on the environment. It is also helpful for developing management plans for the Baltic Sea for the benefit of human populations, but this lack of information hampers policymaking and the formulation of those management plans, such as in the BSAP, particularly for organisations originally focussed on conservation. More work is needed, therefore, to draw together these different facets of health and well-being and ecosystem services. This will ensure the resilience of both the management of the Baltic Sea ecosystems services and the human populations that rely on them.

## Conclusions

The map highlights the lack of evidence that explicitly shows how the consumptive or non-consumptive use of Baltic Sea ecosystem services impacts human health and well-being. This map shows that additional research is needed to ensure plans to protect the Baltic Sea ecosystem include the health and well-being of those exposed to it.

### Implications for Baltic Sea management and policy

Evidence that links ecosystem services with health and well-being would strengthen the calls to protect the environment or to use it sustainably. Linking the evidence for the benefits and risks arising from the ecosystem services that the Baltic Sea provides to health and well-being will improve the knowledge available to tackle management issues arising. The evidence could also be used to inform the public to justify management regimes and increase the acceptability of restrictions where necessary. In addition, the information could be used to educate the public of the values of undervalued systems such as coastal meadows that protects coastlines and provides water purification services. To improve the acceptability of management plans there needs to be evidence to demonstrate that plans will lead to an improvement in health and well-being for the human population, rather than purely dwelling on the deleterious impacts that humans have on the ecosystems.

### Implications for future research (syntheses and primary research)

Primary research is needed to investigate the evidence for the health and well-being impact of marine ecosystem services on human populations. Rather than just noting that there is an increasing potential for human health impact on the Baltic Sea ecosystems, there needs to be an increase in research to quantify such impact. There is also a need to more fully consider the implications in primary research on human populations’ health and well-being rather than solely focussing on the impacts of humans on the environment. Engaging the local populations in the conservation and mitigation efforts needed, however, requires a comprehensive understanding of the values they hold. Management plans would be more readily accepted if the harm or the benefit that human populations faced was more fully understood. This would help society to understand that the range of possible solutions and mitigation measures can benefit both human populations as well as the environment.

Many of the articles in the wider group of articles indicate there are multiple health and well-being risks to human populations. However, periodic multi-disciplinary reviews would provide the necessary linkages to the ecosystem services concept that will support policymaking. Enabling scientifically proven links to be made between the marine ecosystem services with health and well-being arising from the Baltic Sea will lead to a more developed holistic picture that will help to ensure better targeted interventions to address issues.

## Supplementary Information

**Additional file 1.** HWB ROSES for Systematic Map Reports.

**Additional file 2.** List of policies with an impact on the Baltic Sea.

**Additional file 3.** Search strings for all sources.

**Additional file 4.** Excluded articles.

**Additional file 5.** Included articles.

**Additional file 6.** Publication types and journals.

**Additional file 7.** Cluster analysis Figure 12.

**Additional file 8.** Heatmap_Figure 13.

**Additional file 9.** Heatmap_Figure 14.

**Additional file 10.** Cluster analysis Figure 15.

**Additional file 11.** Heatmap_Figure 16.

**Additional file 12.** Heatmap_Figure 17.

## Data Availability

Data available in the additional files.
