# Supplementary material for: Evidence on the impact of Baltic Sea ecosystems on human health and well-being: a systematic map
Source: Environ Evid. 2021 Nov 6;10(1):30. doi: 10.1186/s13750-021-00244-w (PMC8572082; doi:10.1186/s13750-021-00244-w)
Supplement: Supplementary file 1 — Additional file 1. HWB ROSES for Systematic Map Reports. [file 13750_2021_244_MOESM1_ESM.pdf]

Section/sub-section	Topic	Description
Title	Title	The title must indicate that it is a systematic map, and should indicate
Type of review	Type of review	Select one of the following types of review: systematic map,
Authors' contacts	Authors' contacts	The full names, institutional addresses and email addresses for all
Abstract	Structured summary	The abstract of the manuscript must not exceed 500 words and must
Background	Background	Describe the rationale for the review in the context of what is already
Stakeholder engagement	Stakeholder engagement	The actual role of stakeholders throughout the review process (e.g. in
Objective of the review	Objective	Describe the primary question and secondary questions (when
	Definition of the question	Provide reference to the question key elements, e.g. population(s),
Methods	Protocol	Provide citation, DOI or open-access link to published protocol.
	Deviations from protocol	Describe any ways in which the final methods of the review deviate
Searches	Search strategy	Detail the search strategy used, including: database names accessed,
	Search string	Provide Boolean-style full search string and state the platform for
	Languages - bibliographic	List languages used in bibliographic database searches
	Languages – grey literature	List languages used in organisational website searches and web-based
	Bibliographic databases	Provide the number of bibliographic databases searched
	Web-based search engines	Provide the number of web-based search engines searched
	Organisational websites	Provide the number of organisational websites searched
	Estimating comprehensiveness	Describe the process by which the comprehensiveness of the search
	Search update	Describe any update to searches undertaken during the conduct of the
Article screening and study	Screening strategy	Describe the methodology for screening articles/studies for relevance.
	Inclusion criteria	Describe the inclusion criteria used to assess relevance of identified
Critical appraisal	Critical appraisal strategy	Describe here the method used for critical appraisal of study validity
	Critical appraisal used in	Describe how the information from critical appraisal was used in
Meta-data extraction and	Meta-data extraction and	Describe the method for meta-data extraction and coding for studies,
	Approaches to missing data	Describe any process for obtaining and confirming missing or unclear
Data synthesis and	Narrative synthesis strategy	Describe methods used for narratively synthesising the evidence base
	Knowledge gap and cluster	Describe the methods used to identify and/or prioritise key knowledge
	Demonstrating procedural	Describe the role of systematic reviewers (who have also authored
Results	Description of review process	Describe the review process including the volume of evidence
	Number of search results	Provide the number of search results from bibliographic databases
	Number of search results after	Provide the total number of search results from bibliographic database
	Full text screening excludes	Additional file containing list of and reasons for full text exclusions.
	Title screening results	Provide the number of articles retained following title screening.
	Abstract screening results	Provide the number of articles retained following abstract screening.

	Title and abstract screening	Provide the number of articles retained following title and abstract
	Retrieval results	Provide the number of articles retrieved at full text.
	Unobtainable articles	Additional file containing list of unobtainable articles.
	Full text screening results	Provide the number of articles retained following full text screening.
	Consistency checking: screening	Results of consistency checking at all stages (screening, meta-data
	Narrative synthesis	Describe the body of evidence identified using figures and tables,
	Systematic map database	Additional file containing meta-data and coding for included studies.
Discussion	Discussion	Discuss the review results and suggest further enquiry or analysis (e.g.
	Limitations of the review	Discuss possible limitations in the methods used.
	Limitations of the evidence base	Discuss possible limitations in the evidence base.
Conclusions	Knowledge gaps and clusters	Describe knowledge gaps (unrepresented or underrepresented
	Implications for	Summarise the state of the evidence base and discuss the way in
	Implications for research	Discuss the way in which the identified evidence may inform research
Declarations	Competing interests	Describe of any financial or non-financial competing interests that the

References

[1] James, K.L., Randall, N.P. and Haddaway, N.R., 2016. A methodology for systematic mapping in environmental sciences. Environmental Evidence, 5(1

[2] Bayliss, H.R., Haddaway, N.R., Eales, J., Frampton, G.K. and James, K.L., 2016. Updating and amending systematic reviews and systematic maps in env

[3] Haddaway, N.R., Kohl, C., da Silva, N.R., Schiemann, J., Spök, A., Stewart, R., Sweet, J.B. and Wilhelm, R., 2017. A framework for stakeholder engagem

[4] Collaboration for Environmental Evidence. 2018. Guidelines and Standards for Evidence synthesis in Environmental Management. Version 5.0. www

[5] Leeds Institute of Health Sciences. https://medhealth.leeds.ac.uk/info/639/information_specialists/1500/search_concept_tools. Accessed 12/11/20

Further explanation	Checklist/meta-data	Author response
The title should normally be the same or very similar to the review	Meta-data	Evidence on the imp
See CEE Guidance on systematic mapping [1], and on amendments and	Meta-data	systematic map
	Checklist	Yes
	Checklist	Yes
A theory of change and/or conceptual model should be presented that	Checklist	Yes
	Checklist	Yes
The primary question is the main question of the review. The	Checklist	Yes
For other question types see [4,5]	Meta-data	Health and well-bei
The protocol should be peer-reviewed and publicly available online	Meta-data	https://doi.org/1
	Checklist	Yes
	Checklist	Yes
	Meta-data	Geographical
	Meta-data	English
	Meta-data	English
	Meta-data	5
	Meta-data	12
	Meta-data	0
	Checklist	Yes
Compulsory (if update performed). A search update is good practice if	Checklist	n/a
	Checklist	Yes
	Checklist	Yes
Optional	Checklist	No
Compulsory if critical appraisal performed	Checklist	n/a
	Checklist	Yes
	Checklist	Yes
	Checklist	Yes
	Checklist	Yes
Reviewers who have authored articles to be considered within the	Checklist	Yes
	Checklist	Yes
This number should not include web-based search engine or	Meta-data	6456
This number should not include web-based search engine or	Meta-data	300
	Checklist	Yes
Optional if screening titles and abstracts together	Meta-data	n/a
Optional if screening titles and abstracts together	Meta-data	n/a

Optional if screening titles and abstracts separately	Meta-data		2141
	Meta-data		1900
	Checklist	Yes	
	Meta-data		460
	Checklist	Yes	
	Checklist	Yes	
	Checklist	Yes	
	Checklist	Yes	
	Checklist	Yes	
	Checklist	Yes	
	Checklist	Yes	
Reviews must not include practical environmental management	Checklist	Yes	
In this section some advocacy for future research on the reviewed topic	Checklist	Yes	
	Checklist	Yes	

l), p.7.
ironmental management. Environmental Evidence, 5(1), p.20.
nent during systematic reviews and maps in environmental management. *Environmental Evidence* , *6* (1), p.11.
.environmentalevidence.org/information-for-authors.
)17.

Comments

ɔact of Baltic Sea ecosystems on human health and well-being: A systematic map.

See the Systematic map protocol

ng studies linked to the human populations within the marine and coastal environment of the Baltic Sea region (population)

also see protocol

Web of Science (Web of Science Core Collection, Timespan=All years (1975-2020) / LANGUAGE: English), MEDLINE, PUBMED, SCOPUS and CAB [illegible]

See Additional File 3

No critical appraisal was performed

See Additional File 4 for complete list

See Additional File 5

Abstracts
