# Supplementary material for: Evidence on the impact of Baltic Sea ecosystems on human health and well-being: a systematic map
Source: Environ Evid. 2021 Nov 6;10(1):30. doi: 10.1186/s13750-021-00244-w (PMC8572082; doi:10.1186/s13750-021-00244-w)
Supplement: Supplementary file 2 — Additional file 2. List of policies with an impact on the Baltic Sea. [file 13750_2021_244_MOESM2_ESM.docx]

Additional file 2. List of policies with an impact on the Baltic Sea.

| **Organisation** | **Agencies** | **Area of operation** | **Year** | **Weblink** | **Comments** |
| --- | --- | --- | --- | --- | --- |
| EU | Directives and conventions | Water Framework Directive | 1980 | <http://ec.europa.eu/environment/water/water-framework/info/intro_en.htm> |  |
|  |  | EU Habitats Directive | 1992 | <http://ec.europa.eu/environment/nature/legislation/habitatsdirective/index_en.htm> | Harbour porpoises require strict protection under this directive |
|  |  | Water Framework Directive - update | 1998 | https://eur-lex.europa.eu/legal-content/EN/TXT/PDF/?uri=CELEX:32013L0039&from=EN | Watch list of emerging pollutants (http://publications.jrc.ec.europa.eu/repository/bitstream/JRC95018/lbna27142enn.pdf) |
|  |  | Bathing Water Directive | 2006 | <https://www.eea.europa.eu/themes/water/europes-seas-and-coasts/assessments/state-of-bathing-water/bathing-water-directives> |  |
|  |  | Marine Strategy Framework Directive | 2008 | <http://ec.europa.eu/environment/marine/eu-coast-and-marine-policy/marine-strategy-framework-directive/index_en.htm> |  |
|  |  | Environmental Quality Standards Directive | 2013 | <https://rod.eionet.europa.eu/instruments/634> |  |
|  |  | Helsinki Convention | 2014 | <http://www.helcom.fi/about-us/convention> | Convention on the protection of the marine environment of the Baltic Sea area, 1992 |
|  |  | Drinking Water Directive | 2015 | <http://ec.europa.eu/environment/water/water-drink/legislation_en.html> |  |
|  | European Food Safety Agency | Ban on Brominated flame retardants | 2009 | <https://www.efsa.europa.eu/en/topics/topic/brominated-flame-retardants> | European Food Standards Agency has a margin of exposure assessment |
|  |  | Multiannual plan for the stocks of cod, herring and sprat in the Baltic Sea and the fisheries exploiting those stocks | 2016 | <https://eur-lex.europa.eu/legal-content/EN/TXT/?uri=CELEX%3A32016R1139> |  |
|  |  | European Food Safety Authority: UNIT on Biological Hazards and Contaminants (BIOCONTAM) | Ongoing | <https://www.efsa.europa.eu/en/panels/biohaz> |  |
|  | European Chemicals Agency | Regulation of chemicals in the environment | 2007 | <https://echa.europa.eu/regulations/reach/understanding-reach> | REACH is a regulation of the European Union, adopted to improve the protection of human health and the environment from the risks that can be posed by chemicals, while enhancing the competitiveness of the EU chemicals industry. |
|  | Helcom | Baltic Sea Joint Comprehensive Environmental Action Programme (JCP) | 1992 | <http://www.helcom.fi/action-areas/industrial-municipal-releases/helcom-hot-spots> | specifies a series of actions to be undertaken at the polluting Hot Spots around the Baltic Sea drainage basin. The most notorious Hot Spots are point sources such as municipal facilities and industrial plants, but the programme also covers pollution from agricultural areas and rural settlements, and sensitive areas such as coastal lagoons and wetlands where special environmental measures are needed. |
|  |  | Marine Protected Areas (in conjunction with Oceana) | 1994 | <http://www.helcom.fi/action-areas/marine-protected-areas> |  |
|  |  | Baltic Sea Action Plan | 2007 | <http://www.helcom.fi/baltic-sea-action-plan> |  |
|  |  | Marine Sediment Extraction | 2015 | <https://portal.helcom.fi/meetings/PRESSURE%203-2015-278/MeetingDocuments/7-2%20HELCOM%20Recommendation%20on%20marine%20sediment%20extraction%20in%20the%20Baltic%20Sea%20area.pdf> |  |
|  |  | State of the Baltic Sea - second Helcom holistic assessment 2011-2016 | 2016 | <http://www.helcom.fi/Lists/Publications/BSEP155.pdf> |  |
|  |  | Pharmaceuticals in the aquatic environment of the Baltic Sea region: A status report | 2017 | <http://www.helcom.fi/Lists/Publications/BSEP149.pdf> |  |
|  |  | Helcom work with Hazardous Substances | 2018 | <http://www.helcom.fi/baltic-sea-trends/hazardous-substances> | GROUPS AND PROJECTS: PRESSURE Group, Reduction Scheme Core Drafting Group, Sixth Baltic Sea Pollution Load Compilation, HELCOM Pollution Load User System |
|  |  | The Baltic Sea Regional Nutrient Recycling Strategy | 2018 | <https://ccb.se/wp-content/uploads/2018/10/thaaranen_helcom_bsr_nutrient_recycling_strategy.pdf> |  |
| Oceana | International | International organisation working to protect oceans |  | <https://oceana.org/about-oceana/about-us> |  |
|  | Oceana: Baltic | Working on the Marine Protected areas (also HELCOM) |  | http://baltic.oceana.org/en/bl/about-us# |  |
| UN | UNESCO | Working with HELCOM on pharmaceuticals |  |  |  |
|  | Foundation for Environmental Education | Blue Flag awards | 1987 | <http://www.blueflag.global/our-programme/> | Clean Beach Awards |
|  | International Maritime Organisation | International Convention for the Safety of Life at Sea (SOLAS), 1974 | 1974 | <http://www.imo.org/en/About/Conventions/ListOfConventions/Pages/Default.aspx> |  |
|  |  | International Convention for the Prevention of Pollution from Ships, 1973, & Protocol of 1997 (MARPOL) | 1997 |  |  |
|  |  | International Convention on Standards of Training, Certification and Watchkeeping for Seafarers (STCW) & 1995 and 2010 Manila Amendments | 2010 |  |  |
|  |  | Control of ships' ballast water | 2004 | [International Convention for the Control and Management of Ships' Ballast Water and Sediments, 2004](http://www.imo.org/en/About/Conventions/ListOfConventions/Pages/International-Convention-for-the-Control-and-Management-of-Ships%27-Ballast-Water-and-Sediments-(BWM).aspx) |  |
|  |  | Control of sewage from ships | 2011 | <http://www.imo.org/en/OurWork/Environment/PollutionPrevention/Sewage/Pages/Default.aspx> |  |
|  | United Nations Framework Convention on Climate Change | Paris Agreement | 2018 | https://unfccc.int/process-and-meetings/the-paris-agreement/the-paris-agreement |  |
|  | AEWA | Agreement on the conservation of African-Eurasian Migratory Birds | 1996 | [https://www.unep-aewa.org](https://www.unep-aewa.org/) |  |
| ICES | International Council for the Exploration of the Sea | Monitoring of Bycatch of Protected Species in conjunction with HELCOM | 2017 | <http://www.ices.dk/sites/pub/Publication%20Reports/Expert%20Group%20Report/acom/2017/WGBYC/wgbyc_2017.pdf> |  |
|  |  | Strategic Plan | 2019 | <https://issuu.com/icesdk/docs/ices_stategic_plan_2019_web> | ICES commits to a better understanding marine ecosystems and securing the benefits that people derive from them. The purpose of this plan is to de ne our direction and priorities relating to science, data, and advice, and to develop the capacity needed to fulfil this commitment. |
| Local | Municipalities/ Member states | Planning for wind farms, harbour activities, underwater cables, pipelines, construction of buildings, dredging? Sand and gravel extraction? |  |  |  |
| Sources of additional information |  | Report/Document/Webpage |  | Weblink | Comments |
| Oceana |  | The Quark |  | http://oceana.org/sites/default/files/euo/OCEANA_The_Quark_6.pdf | The quark is a section of the Baltic Sea mentioned in Helcom report |
|  |  | Fisheries: Baltic |  | <http://baltic.oceana.org/en/bl/fisheries> |  |
