# Supplementary material for: Evidence on the impact of Baltic Sea ecosystems on human health and well-being: a systematic map
Source: Environ Evid. 2021 Nov 6;10(1):30. doi: 10.1186/s13750-021-00244-w (PMC8572082; doi:10.1186/s13750-021-00244-w)
Supplement: Supplementary file 6 — Additional file 6. Publication types and journals. [file 13750_2021_244_MOESM6_ESM.docx]

Additional file 6

Publication types of the included full-text references (n=67).


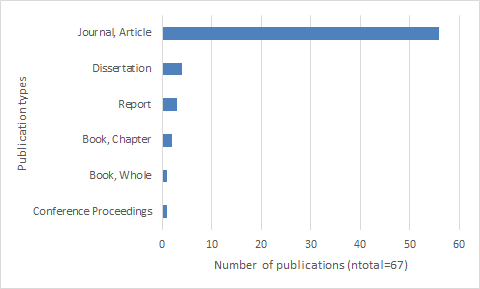


Journals by name

| **Journal** | **No.** |
| --- | --- |
| Aquatic Conservation: Marine and Freshwater Ecosystems | 1 |
| Baltic Coastal Ecosystems | 1 |
| Baltic Sea Environment Proceedings ; 116 B | 1 |
| Biological Conservation | 1 |
| Conservation, Frontiers in Environmental Science | 1 |
| Continental Shelf Research | 1 |
| Ecological Complexity | 1 |
| Ecological Economics | 1 |
| Ecological Indicators | 1 |
| Ecology and Society | 1 |
| Ecosystem Services | 1 |
| Environment International. 2020;137 | 1 |
| Environmental Research Letters | 1 |
| European Journal of Protistology | 1 |
| Forests | 1 |
| Frontiers in Environmental Science | 1 |
| Global Environmental Change-Human and Policy Dimensions | 1 |
| International Journal of Biodiversity Science, Ecosystem Services & Management / Marine & Coastal Ecosystem Services | 1 |
| Journal for nature conservation | 1 |
| Journal of Applied Ecology | 1 |
| Journal of Ecological Engineering | 1 |
| Journal of Environmental Economics and Policy | 1 |
| Landscape Ecology | 1 |
| Managing a Sea | 1 |
| Marine Ecosystem Ecology - Frontiers in Marine Science | 1 |
| Maritime Spatial Planning in the Baltic Sea Region and Way Forward. Riga, VASAB | 1 |
| Oceanologia | 1 |
| PloS one | 1 |
| Population Ecology | 1 |
| Proceedings of the National Academy of Sciences of the United States of America | 1 |
| Science of the Total Environment | 1 |
| Sustainability | 1 |
| Water Economics and Policy | 1 |
| Ecosystems | 2 |
| Environmental & Resource Economics | 2 |
| Journal of Coastal Conservation | 2 |
| Journal of Environmental Management | 2 |
| Marine Policy | 2 |
| Ocean & Coastal Management | 2 |
| Journal of Coastal Research | 3 |
| Thesis -BSc | 4 |
| Report | 5 |
| Ambio | 10 |
