# Supplementary material for: Evidence on the impact of Baltic Sea ecosystems on human health and well-being: a systematic map
Source: Environ Evid. 2021 Nov 6;10(1):30. doi: 10.1186/s13750-021-00244-w (PMC8572082; doi:10.1186/s13750-021-00244-w)
Supplement: Supplementary file 8 — Additional file 8. Heatmap_Figure 13. [file 13750_2021_244_MOESM8_ESM.html]

EPPI-Mapper


X

- Filters
- Hide Headers
  Show Headers
- Fullscreen
  Exit Fullscreen
- About
- Submit a Study
- View Records

Hover your mouse over the bubbles to show the number of articles represented

Click on the bubbles to show the article details

Generated using v.2.1.0 of the EPPI-Mapper
powered by EPPI Reviewer
and created with


by the
Digital Solution Foundry team.
